# In-scanner thoughts contribute to resting-state functional connectivity

**DOI:** 10.1038/s41467-026-74953-6

**Published:** 2026-06-27

**Authors:** Javier Gonzalez-Castillo, Megan A. Spurney, Ka Chun Lam, Isabel S. Gephart, Francisco Pereira, Daniel A. Handwerker, Julia W. Y. Kam, Peter A. Bandettini

**Affiliations:** 1 Section on Functional Imaging Methods, NIMH, NIH, Bethesda, MA USA; 2 Department of Psychology, Northwestern University, Evanston, IL USA; 3 Machine Learning Team, NIMH, NIH, Bethesda, MA USA; 4 Department of Psychology, University of Calgary, Calgary, AL Canada; 5 Hotchkiss Brain Institute, University of Calgary, Calgary, AL Canada; 6 Functional MRI Core, NIMH, NIH, Bethesda, MA USA

**Keywords:** Consciousness, Cognitive neuroscience, Biomarkers

## Abstract

Resting-state fMRI (rsfMRI) scans—acquired in the absence of experimentally controlled stimuli or task demands—are widely used to identify aberrant patterns of functional connectivity (FC) in clinical populations. To minimize interpretational uncertainty, researchers routinely control for across-cohort disparities in age, gender, comorbidities, and head motion. Yet, studies rarely consider the possibility that systematic differences in inner experience (i.e., how subjects think and feel during the scan) directly affect FC measures. Here, using an rsfMRI dataset comprising 469 scans with retrospective experiential annotations, we show that summary descriptors of in-scanner experience are reproducible across visits and subject-specific, consistent with trait-like characteristics. We further show that widespread significant differences in FC are observed between scans that are associated with different reported experiential profiles, and that FC can predict specific experiential dimensions with performance comparable to that reported for demographic, cognitive, and clinical variables. Together, these findings highlight the key role that in-scanner experience should play when interpreting FC in the context of rsfMRI. Given that the available experiential measures are retrospective summaries, these results speak to stable experiential tendencies rather than potential moment-to-moment, state-dependent relationships between ongoing experience and concurrent brain activity.

## Abstract (web summary)

Summary aspects of internal mentation—the form and content of thoughts—occurring during resting-state scans show trait-like qualities, contribute to functional connectivity, and should be considered when interpreting results.

## Introduction

Resting-state scans are a central component of large-scale, international neuroimaging efforts aimed at mapping the human functional connectome in vivo, in both healthy and patient populations^1^. This prominence of resting-state in cognitive and clinical neuroscience research is motivated by its experimental simplicity, short scanning time (which translates into cost effectiveness), richness of information, and low demands on participants (typical instructions might be “*remain still and awake as you let your mind wander freely”)*. Yet, despite its wide adoption, we still lack an understanding of the nature and biological purpose of the neural processes that give rise to the observed synchronized patterns of spontaneous activity during rest^2,3^. This knowledge gap severely limits both our ability to interpret results and to develop reliable brain-based biomarkers of disease.

Neural mechanisms previously suggested as explanatory phenomena for resting-state functional connectivity patterns (rsFC) include memory consolidation^3^, learning^4,5^, fluctuations in wakefulness^6,7^, homeostatic processes needed to maintain the brain’s functional integrity^3^, the replay and update of predictive models of the environment^8^, processing of interoceptive signals from the rest of the body^9^, and those associated with different aspects of the subjective experience participants undergo while being scanned^2,10,11^. Of these, the role of the in-scanner subjective experience is perhaps the least established^12^. This is because researchers rarely collect information about what participants think and feel inside the scanner. A few instances where such data is available suggest that some aspects of in-scanner experience can be related to resting-state signals. For example, reported levels of imagery correlate with the strength of resting-state fluctuations in perilingual cingulate cortex^13,14^ and the face fusiform area^15^, and reported levels of comfort with that of somatosensory cortex^14^; a higher propensity to focus in-scanner thoughts on current concerns (as opposed to future planning) has been linked to stronger betweenness centrality for the left middle frontal gyrus^16^ (a key component of the salience network); increased activity in the dorsal attention network to subjective reports of attentional control^17^; increase activity in lateral fronto-parietal cortices to increases in external awareness^18^; stronger connectivity between the default mode network and the control networks has been linked to reduced variability in the semantic content of thoughts^10^; and the qualia of inner voices (i.e., inner hearing vs. inner speaking) was associated with differential patterns of spontaneous activity during rest in language related areas^19^. There are also studies linking a limited number of brain states (i.e., recurrent resting-state whole brain activity patterns) to the amount of time subjects report solving problems about the future or having intrusive thoughts about the past^20^; as well as to individual variability in FC between the visual and sensorimotor systems^21^.

Despite these initial reports, it is not yet established how strongly systematic differences in in-scanner experience influence rsFC patterns. This matters because unmodeled variance based on these differences could hide aberrant patterns of inter-regional communication linked to clinical symptoms and hinder efforts aimed at developing brain-based biomarkers of disease progression and therapeutic efficacy. In fact, some have argued for the use of naturalistic experiment designs in detriment of resting-state, due to the many interpretational challenges stemming from resting-state lacking a well-defined relationship with cognitive processes^22^. Conversely, others have argued that variations in in-scanner experience might have a minimal or no modulatory effect at all on rsFC^3^. To shed light on these debates, which are key for forecasting the true clinical potential of rsfMRI and understanding the role that in-scanner experience might play in common research applications of rsfMRI, we seek to answer three specific questions.

First, are patterns of in-scanner thoughts during rest characteristic and consistent in individuals over time? If so, putative modulatory effects of in-scanner experience in rsFC will show some level of stability across repeated scans, potentially confounding (or explaining) the relationship between rsFC and other subject-specific, trait-level characteristics of interest. It would also mean that such effects could not be easily removed by simple averaging across successive scanning sessions.

Second, do scans that systematically differ in terms of thought content show significant differences in rsFC? If so, are such differences interpretable and in agreement with our current understanding of the functional organization of the brain? A common way to uncover systems-level FC correlates of brain disease is to acquire resting-state scans in two groups—those afflicted by the condition of interest and healthy controls—and to contrast those after careful pre-processing and matching of samples in terms of head motion and basic demographics. Observed differences that survive strict statistical testing are then interpreted as being related to the symptomatology being examined^23^. Rarely do researchers consider the possibility that such observations, in full or partially, might be explained by systematic differences in in-scanner experience across the groups. To evaluate whether this should be a concern, we take hundreds of scans from a healthy population and subdivide them into sets that differ in terms of thought content, but are otherwise similar regarding sample size, gender, age, head motion, and wakefulness. Should a comparison across these sets render widespread significant differences in rsFC, this will suggest that in-scanner experience should be considered a potential explanatory factor in rsFC studies of populations known to have altered spontaneous thought patterns, which is the case for those afflicted by dysphoria^24^, major depressive disorder^25–27^, autism spectrum disorder^28^ and borderline personality disorder^29^; to name a few.

Third, is it possible to predict different aspects of in-scanner thoughts using rsFC, in a manner similar to how other studies predict phenotypical scores related to personality traits^30^, cognitive abilities^31,32^ and clinical symptoms or labels^33,34^? If so, this would suggest that the imprints of in-scanner experience in rsFC are equivalently strong to those of these other phenotypical characteristics commonly targeted by resting-state research, and can be potential targets for better interpretation of clinically-relevant variations.

Here, we answer these three questions affirmatively, using a subset of 469 resting-state scans from the publicly available Max Planck Institute Mind-Brain-Body dataset^35^. These scans are annotated with retrospective debriefing self-reports about in-scanner thoughts and wakefulness levels (Table 1). Although limited in scope, these annotations provide rare and valuable information about participants’ subjective experience during rsfMRI acquisition. Given the retrospective nature of these experiential measures, however, the present analyses focus exclusively on summary dimensions of in-scanner experience, such as participants’ general tendency to think in images or to engage in inner speech. They do not test whether momentary experiential events, such as imagining a walk through the woods during light rain, are associated with concurrent fluctuations in brain activity. Such state-level questions would require temporally resolved experiential measurements, such as intermittent in-scanner probes^36^ or continuous thought reporting^10^, which are not available in this dataset.

**Table 1:** Experiential Questions following the completion of each resting-state scan

TARGET	LABEL	STATEMENT
CONTENT OF THOUGHTS	Surroundings	I thought about my environment/surroundings
	People	I thought about other people
	Myself	I thought about myself
	Past	I thought about past events
	Future	I thought about future events
	Negative	I thought about something negative
	Positive	I thought about something positive
FORM OF THOUGHTS	Intrusive	My thoughts were intrusive
	Specific	My thoughts were more specific than vague
	Words	My thoughts were in the form of words
	Images	My thoughts were in the form of images
WAKEFULNESS	Wakefulness	I was completely awake

Our results highlight the important role of in-scanner experience as a key explanatory factor for rsfMRI. They also suggest that putative systematic differences in in-scanner thought patterns (e.g., excessive ruminative thoughts, disparate focus of attention, etc.) across surveyed populations can cloud the interpretation of population studies by adding one more source of unmodeled variance. To mitigate these issues and facilitate more detailed interpretations of results, future resting-state studies should consider the inclusion of experiential self-reports as a companion to every scan. This is a relatively simple addition, compared to the cost and complexity of executing a single resting-state scan, and would enrich resting-state datasets with new opportunities for analysis and discovery. For example, experiential measures could be incorporated into statistical models in a manner analogous to other individual-difference variables, depending on the scientific question. Doing so may improve the accuracy and interpretability of rsfMRI-based predictive models and help uncover new brain–behavior relationships. However, as with any covariate, these measures should not be treated as nuisance variables by default. Care is needed to avoid inadvertently removing variance of substantive interest, particularly when specific in-scanner experiential profiles or patterns of thought may be closely related to the clinical phenotype under study (e.g., rumination in depression). Similarly, such richer resting-state datasets would also allow the investigation of the neural correlates of self-generated thought processes such as creative thinking^37^, mind-wandering^38^, interoceptive awareness^39^, intrusive thinking^40^ and mind blanking^41^.

## Results

### Self-report annotations of resting-state scans

**Fig. 1:**
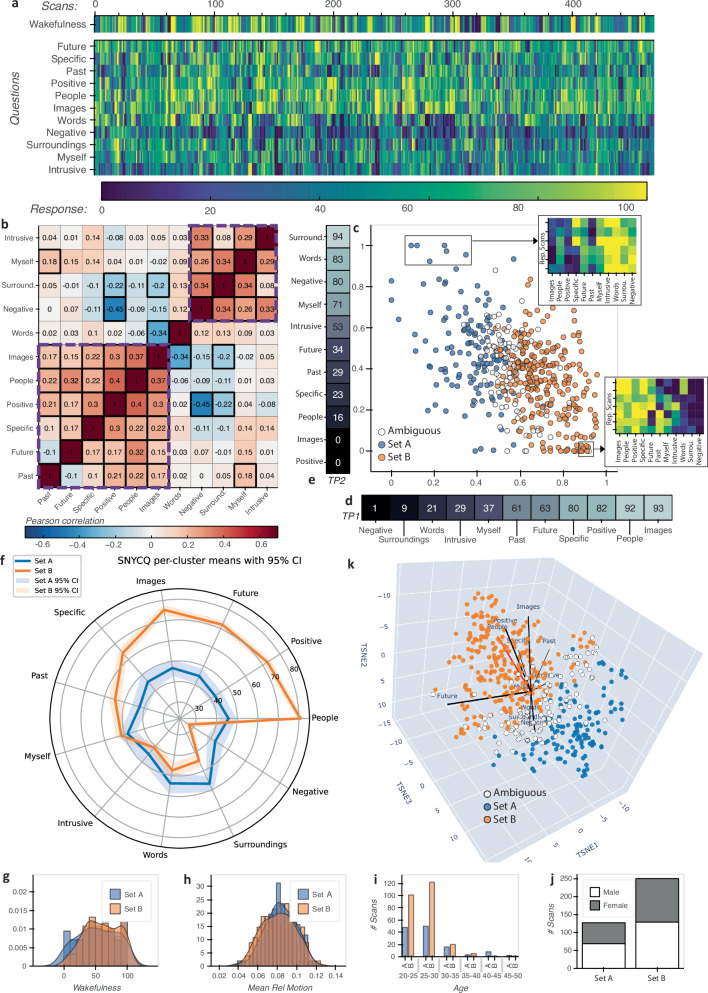
Experiential data and scan bipartition. **a** Responses to the experiential questionnaire (rows) for all scans (columns; see Supplementary Fig. 1 for distribution-based view). Top row is “wakefulness”; matrix below shows responses to the other 11 questions. **b** Correlation between responses to all questions except wakefulness. Significant correlations (two-sided test using the Beta distribution of correlation values implemented in *scipy.stats.pearsonr*, *p*_Bonf_ < 0.05, num comparisons=55) are marked by a bold black outline. Sets of questions (dashed magenta outline) correlate more strongly among themselves. **c** 2D representation of self-report data (except wakefulness) generated with ICQF^43^ (Interpretable Constrained Questionnaire Factorization). Each scan is a colored dot. Axes represent two thought patterns: X-axis (Thought Pattern 1; TP1) represents positive thoughts in the form of images about other people; Y-axis (Thought Pattern 2; TP2) represents thoughts about the surroundings, in the form of words, and negative valence. Dots colored by set membership (GMM). Answers for two sets of scans are depicted to the right. On top, answers for 6 scans with high loadings for TP2 and low loadings for TP1. High responses concentrate on the questions that more heavily relate to TP2 (e.g., negative, surroundings, words, and intrusive). Below that, responses to 7 scans with high loadings for TP1 and low loadings for TP2. Here, higher scores concentrate on the images, people, positive, and specific items of the questionnaire. **d** Relationship between TP1 and questionnaire items as a vector of weights outputted ICQF, which indicates how strongly each questionnaire item relates to this Thought Pattern axis. **e** Same as d, but for TP2. **f** Radar plot showing average values (bold lines) and 95% confidence intervals (shaded region) of sNYCQ items for scan sets A and B (See Supplementary Fig. 2 for distributions). **g** Distribution of wakefulness answers for the two sets **h** Distribution of head motion estimates for the two set. **i** Distribution of age range for both sets. **j** Sex counts in both sets. **k** 3D representation generated with T-SNE^37^. Scans are again colored as in (**c**). Source data are provided as a Source Data file (*n* = 469 scans).

At the end of each resting-state scan, participants filled out the short New York Cognition Questionnaire (sNYCQ, Table 1)—which consists of a subset of questions from the original version of the New York Cognition Questionnaire developed by Gorgolweski et al^13^.—that included seven statements about the content of their thoughts during the scan (i.e., “*I thought about my environment/surroundings*”), four about the form of the thoughts (i.e., “*My thoughts were in the form of images*”), and one about the subjectively perceived level of wakefulness (i.e., “*I was completely awake*”). For each statement, participants responded with a number between 0 (“Describes my thoughts not at all”) and 100 (“Describes my thoughts completely”) (Fig. 1a; see Supplementary Fig. 1 for distributions). Because the modulatory role of wakefulness on rsFC is already well-stablished^6,7,42^, we kept the answers to the wakefulness statement separate from the rest of the questionnaire during the analyses.

Figure 1b shows the correlation between responses to items about the form and content of thoughts, which suggests the presence of latent axes of variability (dashed rectangles). To further assess this initial observation and determine whether the sample could be meaningfully subdivided into two separate sets based on reported experience, we conducted clustering and dimensionality-reduction analyses on the experiential data.

We applied two clustering algorithms—K-means and Gaussian Mixture Modeling (GMM)—and assessed the consistency, quality, and stability of the resulting partitions for k = 2. The adjusted Rand index (ARI) between the two methods was 0.72, indicating substantial agreement and that the identified subdivisions were stable across methods. We then computed the Silhouette Index (SI), a measure of clustering quality. The SI was 0.162 for GMM and 0.154 for K-means. Although these values indicate that the data do not form two fully separated clusters and may instead lie along a continuum, they nonetheless support the interpretation that the sample can be viewed as comprising two distinguishable subsets. The slightly higher SI for GMM also suggests that GMM provides a better fit for these data. Finally, we assessed the robustness of the GMM clustering solution using a bootstrapping analysis (10,000 permutations; 80% subsampling rate). The ARI across iterations was 0.83 ± 0.08 (mean ± stdv), indicating high consistency of the bipartition. In summary, the clustering analyses show that—while fully independent clusters are not present—the sample can be reliably subdivided into two subsets, and this subdivision is stable across subsampling procedures and clustering algorithms.

Next, the experiential data were submitted to two dimensionality reduction algorithms: Interpretability Constrained Questionnaire Factorization (ICFQ)^43^ and T-Stochastic Neighbor Embedding (T-SNE)^44^. T-SNE is a widely adopted method in the machine learning literature, yet T-SNE dimensions are difficult to interpret. ICQF, although less established, is explicitly designed to generate easily interpretable (e.g., no negative weights) latent dimensions. Figure 1c shows the experiential data in the 2D space estimated using ICQF. Here, each scan is represented by a point colored according to set membership as identified with GMM (k = 2). Scans are assigned to Sets A (blue; 127 scans) or B (orange; 250 scans) only if their probability membership is above 0.8; otherwise, scans are marked as ambiguous (white; 92 scans). We observe good separation of the sets in this 2D view. The same is true for the 3D view generated with T-SNE (Fig. 1k).

Because ICQF links original questionnaire items to latent dimensions using only positive weights, the axes in Fig. 1c can be interpreted as denoting two overall forms of thought (i.e., “*Thought Patterns*”) present in the data. Moreover, because loadings on these axes are constrained to values between 0 and 1, the location of a scan in this 2D space represents how much each of these two Thought Patterns is associated with a given scan. Larger weights on the X-axis (Thought Pattern 1 or TP1) indicate scans strongly associated with thoughts in the form of images, about other people and with a positive valence (Fig. 1d). Larger weights on the Y-axis (Thought Pattern 2 or TP2) indicate scans associated with thoughts focused on the surroundings, self, of negative valence, and in form of words (Fig. 1e). The same associations between scan sets and sNYCQ items are observed if, instead of interpreting the data in terms of TP1 and TP2, one directly compares the distribution of sNYCQ items across the two sets (Fig. 1f, Supplementary Table 1; and Supplementary Fig. 2).

Defining these two sets is a first step towards answering the first two questions targeted in this work: 1) do participants consistently revert to similar thought patterns when asked to rest inside the scanner? and 2) do systematic differences in thought pattern lead to significant differences in rsFC?

### Stability of in-scanner experience across sessions

**Fig. 2:**
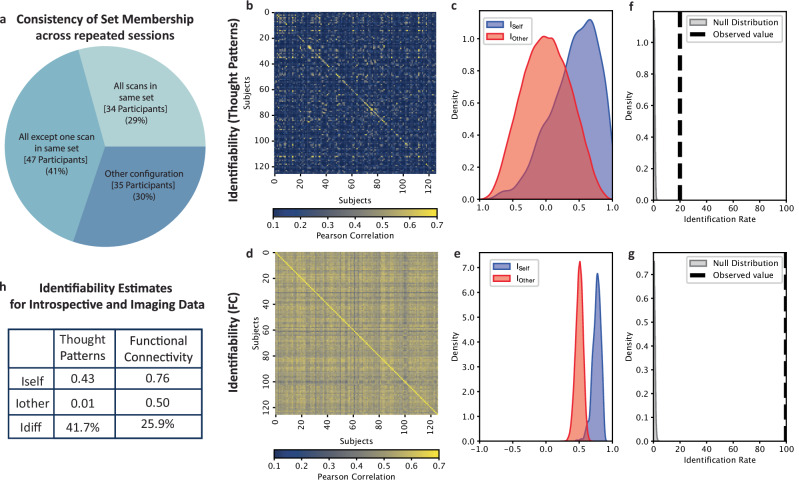
Identifiability properties of thought patterns. **a** Pie plot showing how often do scans from the same participant belong to the same scan set (i.e., A, B or ambiguous). **b** Identifiability matrix for introspection data. **c** Distribution of I_Self_ and I_Other_ values for the introspective data. **d** Identifiability matrix for the functional connectivity data. **e** Distribution of I_Self_ and I_Other_ values for the functional connectivity data. **f** Identification rate for the introspection data (dashed vertical line) and null distribution for 10,000 permutations. **g** Identification rate for the functional connectivity data (dashed vertical line) and null distribution. **h** Average identifiability estimates for both experiential data (left) and imaging data (right). Source data are provided as a Source Data file (*n* = 462 scans).

One way to reformulate the first question in terms of the sets we just defined is: how often do scans from the same subject belong to the same set? If we consider only scans for subjects who completed a minimum of three resting-state scans (442 scans across 116 subjects), we observe that for 29% of those participants, all scans belong to the same set; for 41% all scans except one are in the same set; and only for 30% of participants scans are either evenly distributed across two sets or separated across the three sets (Fig. 2a). This result suggests that participants tend to, more consistently than not, have thoughts of similar characteristics across consecutive resting-state scans.

To further evaluate if the overall content and form of thoughts during rest reflect trait-like qualities in subjects, we estimated two identifiability metrics that do not rely on clustering: differential identifiability^45^ and identifiability rate^31^. We did this, separately, for the experiential and the imaging data using scans from subjects with at least two visits. Figure 2b shows the identifiability matrix for the experiential data, and Fig. 2d for the FC data. Both matrices show a clear diagonal, suggesting that within-subject similarity is greater than across-subject similarity for both data types. This is further confirmed by the distributions (Fig. 2c,e) and average values (Fig. 2h) of self-identifiability (I_Self_), others’ identifiability (I_Other_), and differential identifiability (I_Diff_).

Finally, identification rate^31^ was also computed for both data types, and their significance was assessed against 10,000 random permutations (Fig. 2f,g). The identification rate for introspective data was 19.9%, and that of FC data 99.8%. In both cases, the identification rate was statistically significant.

### Differences in functional connectivity due to systematic differences in thought patterns

To evaluate the second question concerning whether systematic differences in thought patterns translate into significant differences in FC, we focused our attention on the scans in the two sets *(A* and *B*), as the above analyses show that they systematically differ in terms of reported thought patterns.

**Fig. 3:**
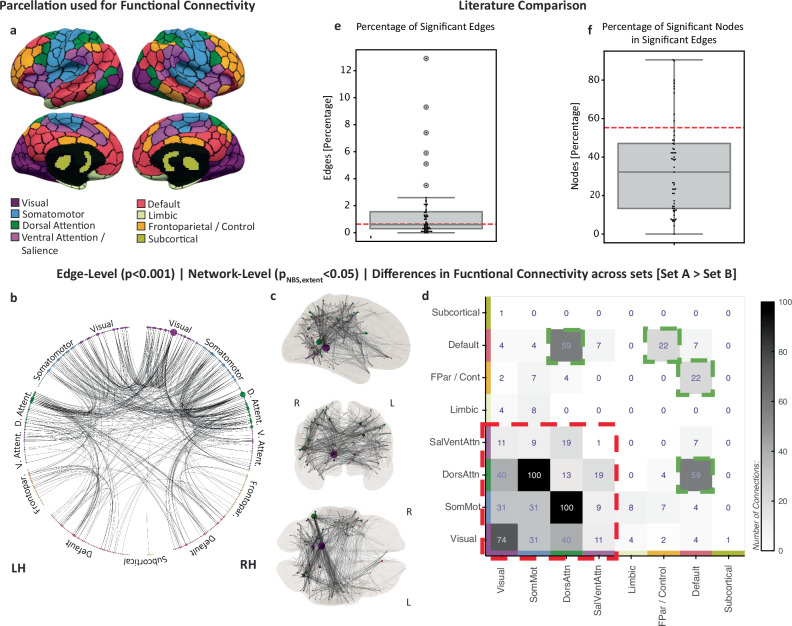
Significant differences in FC across scans with different thought patters. **a** Schematic of the parcellation used for the generation of FC matrices. We relied on the 400 ROI Shaeffer atlas organized into 7 different networks, as well as a set of additional subcortical ROIs from the AAL atlas. **b** Circos plot with connections rendered as significant (edge-level Threshold *p* < 0.001, then NBS^48^ corrected for network extent at *p* < 0.05, 5000 permutations, two-sided) for the Set A *> Set B* contrast. In this plot, ROIs are depicted as colored dots (color indicating network membership) on a circumference, and significant connections are depicted as black lines. ROIs are grouped by network and hemisphere, with left hemisphere ROIs on the left, and right hemisphere ROIs on the right of the plot. The size of the dot is proportional to the degree of the ROI (i.e., number of significant connections emerging from the ROI). **c**, Same information as in b, but this time on a glass brain. ROI size denotes the degree, and ROI color denotes network membership. **d** Counts of between- and within-network connections significant for the contrast *Set A > Set B*. **e** Distribution of percentage of significant edges reported in the NBS literature (see Methods for details on how manuscripts entering these analyses were selected; *n* = 59). **f** Distribution of percentage of nodes contributing to significant edges in the same literature. In both **e** and **f**, boxplots show the distribution across the examined literature, and the red dashed line shows the value observed in this work. The center of the boxplox represent the median, the box extends from the first quartile to the third quartile, whiskers extend to show the rest of the distribution, and black dots represent values for individual contrasts in the literature entering the distribution. Those corresponding to outliers are marked with an outer light gray ring. Source data are provided as a Source Data file (Set A: *n* = 127 scans; Set B: *n* = 250 scans).

FC matrices for all scans in the *A* and *B* sets were created using the 400 ROI/7 network Schaefer Atlas^46^, modified to also include 8 subcortical ROIs from the AAL Atlas^47^ (Fig. 3a, Supplementary Fig. 3). Significant differences in rsFC across these two sets were evaluated using Network-Based Statistics^48^ (NBS; *p*_*NBS*_ < 0.05). We found 458 connections (0.64% of all possible connections) involving 210 nodes (59%) to be significantly stronger for *Set A* as compared to *Set B* (Fig. 3b–d). These connections tend to be lateralized to the right hemisphere (${{FC}}_{{laterality\_index}}=-0.32$). No significant connections were found for the inverse contrast. Counts of connections at the network level reveal that the larger proportion of significantly stronger connections correspond to those involving nodes of both the Dorsal and Ventral Attention networks and primary unimodal networks (dashed red square in Fig. 3d). Similarly, we also observe a second set of significantly different connections involving the Default Mode Network and the Fronto-Parietal Control Dorsal Attention Network (green dashed squares in Fig. 3d). Because NBS results can be sensitive to the choice of edge-level threshold, we repeated the analyses using a less restrictive edge-level threshold of *p* < 0.005, while keeping the network-level threshold unchanged (*p*_NBS_ < 0.05). This threshold yielded a more significant component, comprising 2,339 connections across 342 nodes. Despite this increase, the distribution of significant connections across the eight networks was qualitatively similar to that observed in the primary analysis (Supplementary Fig. 7). The laterality index also remained negative, consistent with right-lateralized effects under this less restrictive edge-level threshold (FC_laterality index_ = −0.27).

Because both wakefulness^6,7,42^ and head motion^49^ are known to strongly modulate rsFC, we checked for differences in these two confounds across the two sets. Figure 1g shows the distribution of responses to the wakefulness question across the two sets. No significant difference was observed (Fig. 1g; *Mann-Whitney U* = *13978, p* = 0.06). Figure 1h shows the same information for mean head motion estimates. No significant difference was observed (*Mann-Whitney U Test* = 17039*, p* = 0.24). Additionally, we also checked for differences in demographics. Figure 1i shows scan counts per age range. No significant difference was observed (*Wilcoxon Test* = *5, p* = 0.31). Finally, Fig. 1j shows scan counts by sex. Although not exactly matched across sets, both sets contain similar distributions of male and female subjects.

Next, to evaluate how our results compare to prior clinical work that relies on NBS to uncover clinically relevant rsFC patterns, we calculated the percentage of significant edges (or connections) and nodes (or ROIs) contributing to those in a set of 50 recent scientific papers (see Methods section for details on how these were selected and Supplementary Table 6 for the list of manuscripts). Figure 3e shows the distribution of the percentage of significant edges in the selected manuscripts as a box plot. Our results (0.63%) are marked as a dashed red line. Figure 3f shows equivalent information in terms of the percentage of nodes contributing to significantly different connections. Both figures demonstrate that systematic differences observed in thought patterns can induce significant differences in FC that have spatial extent like that previously reported when comparing healthy and clinical populations.

**Fig. 4:**
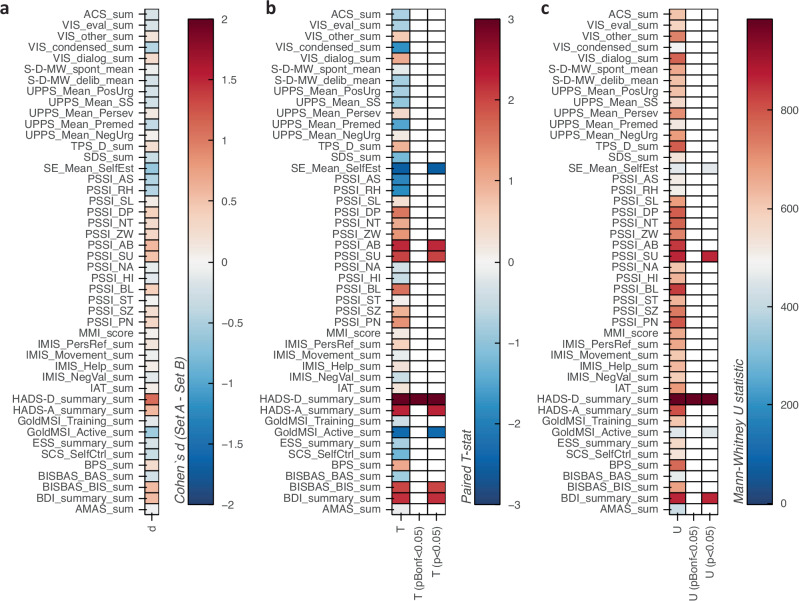
Differences in externally measured behavioral traits across scan sets. **a** Cohen’s d (Set A – Set B) for all the behavioral metrics listed in Supplementary Table 2 (please check table for abbreviations). **b** Statistically significant differences across sets (Set A vs. Set B) according to independent T-tests (two-sided). **c** Statistically significant differences across sets (Set A vs. Set B) according to Mann-Whitney Test (two-sided). Source data and number of samples entering each test are provided as a Source Data file.

Despite all participants having no history of psychiatric or neurological disorders, the assignments being based solely on reported in-scanner experience, and the sets not differing systematically in head motion, wakefulness, or basic demographics, it remains possible that individuals in each set varied along subclinical or phenotypic traits not previously considered. To examine this possibility, we tested for between-set differences across 46 behavioral metrics collected outside the scanner (see Supplementary Tables 2 and 9 for details). Figure 4 summarizes these results. Eight behavioral metrics passed an uncorrected threshold (*p* < 0.05) in the T-tests, and five did so in the Mann-Whitney test. However, only one measure—HADS-D—survived Bonferroni correction in both analyses. HADS-D is a subscale of the Hospital Anxiety and Depression Scale (HADS)^50^ that assesses subclinical depressive symptoms over the week preceding the assessment.

### Prediction of self-reports based on Functional Connectivity

We used the Connectome-based Predictive Modeling (CPM) framework^51^ to evaluate the third question concerning whether it is possible to predict different aspects of in-scanner experience based on rsFC. Using data from all 469 scans (two outlier scans removed), we attempted to predict wakefulness levels, factor scores on TP1 and TP2, and individual responses to the 11 questionnaire items about the form and content of in-scanner thoughts. Prediction accuracy was evaluated in terms of Pearson’s correlation between observed and predicted values after motion orthogonalization. Significance of the correlation scores was evaluated non-parametrically against null distributions computed on 10,000 randomizations (see Methods for additional details).

**Fig. 5:**
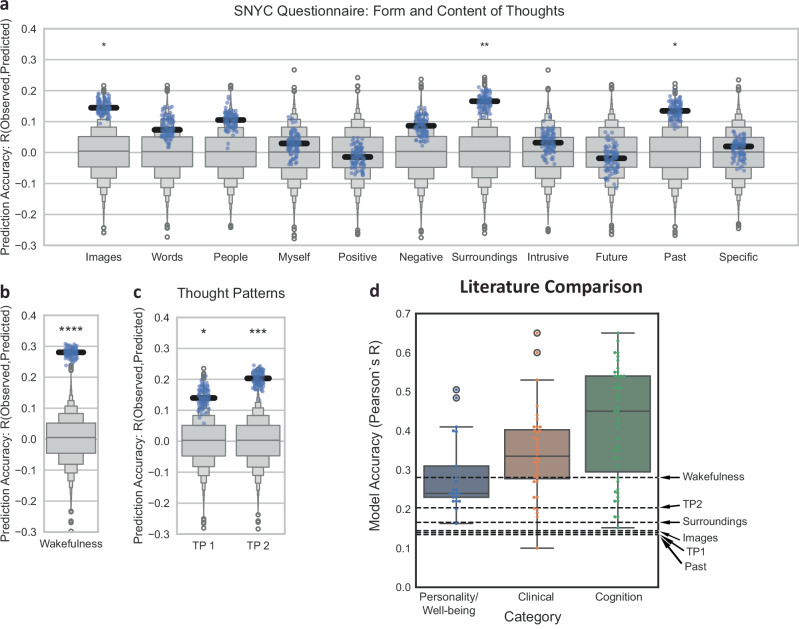
Prediction accuracy for subject-level cross-validation Connectome Predictive Modeling (CPM) analyses (*n* = 469 scans). **a** Answers to the 11 items about the form and content of in-scanner thoughts. **b** Answers to item *“I was completely awake”*. **c,** *Thought Pattern 1* (TP1) and *Thought Pattern 2* (TP2) as defined using the Interpretability Constrained Questionnaire Factorization (ICQF) algorithm. In all plots, the gray boxenplot depicts the null distribution of accuracy values across 10,000 randomized permutations. Blue dots correspond to the Pearson’s correlation between observed and predicted values (i.e., prediction accuracy) in each of the 100 iterations used to predict each target. Bold black lines represent the median prediction accuracy across the 100 iterations. Significance annotations correspond to the non-parametric, one-sided, uncorrected *p*-value of the median prediction accuracy according to each null distribution (^****^ *p* < 0.0001, ^***^ *p* < 0.001^, **^ *p* < 0.01, ^*^ *p* < 0.05). For exact *p*-values, see Supplementary Table 3. **d**, Comparison to previously published studies that used CPM to predict cognitive (*n* = 43), personality (*n* = 36), or clinical traits (*n* = 21) based on full-brain functional connectivity. For each category of studies, we provide a boxplot depicting the distribution of reported accuracy values (center represents the median, colored boxes extend from the first quantile to the third quantile, whiskers extend to show the rest of the distribution, except outliers shown as diamonds). Colored dots represent all individual accuracy values contributing to each distribution. In addition, horizontal dashed black lines show accuracy for the 6 models rendered significant in this study. Details on how we conducted the literature search contributing to this panel can be found in the online methods. Supplementary Table 7 list the studies contributing to this figure panel. Source data are provided as a Source Data file.

As the dataset includes repeated scans from the same participants, our primary analyses used subject-level cross-validation folds. Specifically, all scans from a given participant were assigned to the same fold within each cross-validation iteration, ensuring that no participant contributed scans to both the training and test sets. Under this more conservative procedure, CPM significantly predicted reported wakefulness levels, TP1 and TP2 factor scores, and responses to the Images, Surroundings, and Past questionnaire items (*p*_*non-parametric, uncorrected*_ < 0.05; Fig. 5a–c; Supplementary Table 3). Of these, “Past” did not survive multiple comparison correction based on the False Discovery Rate (FDR^52^) method (*p*_*non-parametric*,FDR_ < 0.05).

For comparison, we also repeated the analyses using scan-level cross-validation folds, in which subject identity was not constrained, and scans from the same participant could appear in both the training and test sets. Under this less conservative procedure, all 14 experiential indices were significantly predicted from rsFC (Supplementary Fig. 4). Because this approach allows subject-specific information to contribute to prediction accuracy, we do not interpret these secondary results as additional evidence that rsFC contains information about in-scanner experience. Rather, they illustrate how dependencies among repeated observations from the same individuals can inadvertently inflate the predictive performance of traits. Accordingly, all subsequent results regarding successful CPM models correspond to the subject-level cross-validation analyses.

**Fig. 6:**
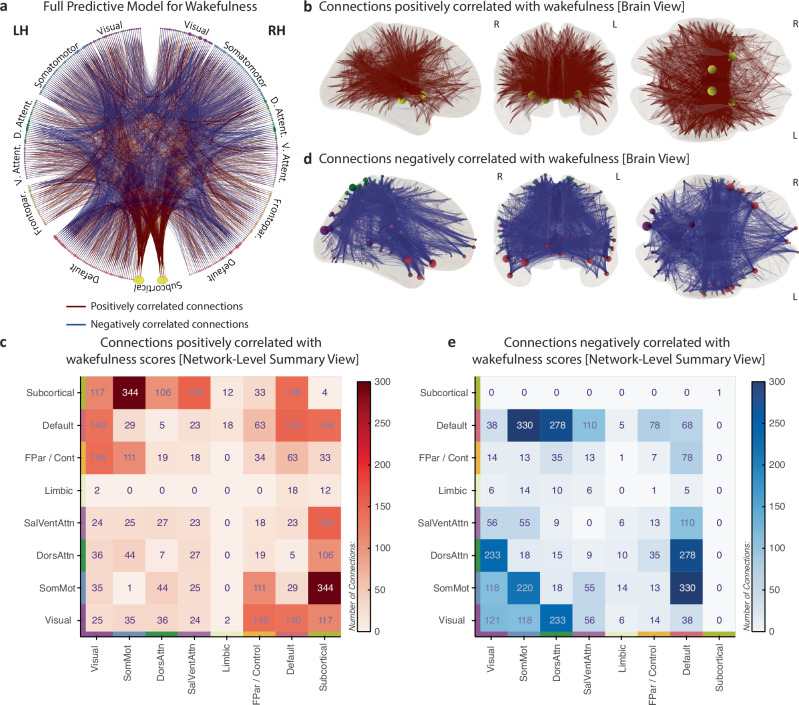
Subject-level cross-validation CPM (Connectome Predictive Modeling; n = 469 scans) model for Wakefulness item “I was completely awake”. **a** Circos plot depicting connections found to correlate positively (dark red) and negatively (dark blue) with the prediction target. **b** Connections positively correlated with reported levels of Wakefulness on a glass brain. **c** Counts of within- and across-network connections that correlate positively with reported levels of Wakefulness. **d** Connections negatively correlated with reported levels of Wakefulness on a glass brain. **e** Counts of within- and across-network connections that correlate negatively with reported levels of Wakefulness. In **a**, **b**, and **d**, the size of the circles representing regions of interest (ROI) is proportional to the number of connections that include the ROI. The color of the circles depicts network membership. Source data are provided as a Source Data file.

To predict Wakefulness, subject-level cross-validation CPM relied on a model that includes 3800 connections (5.3% of all possible connections) distributed across all seven networks (Fig. 6a): 1828 (2.5%) of these connections exhibit positive correlation with the prediction target, and the remaining 1872 (2.6%) show negative correlation. A large fraction of the positively correlated connections represent links between subcortical and cortical regions (Fig. 6b, c). Conversely, connections that are negatively correlated with Wakefulness are cortex-to-cortex connections involving primarily the Default Mode, Ventral and Dorsal Attention, and primary unimodal networks (Fig. 6d, e).

**Fig. 7:**
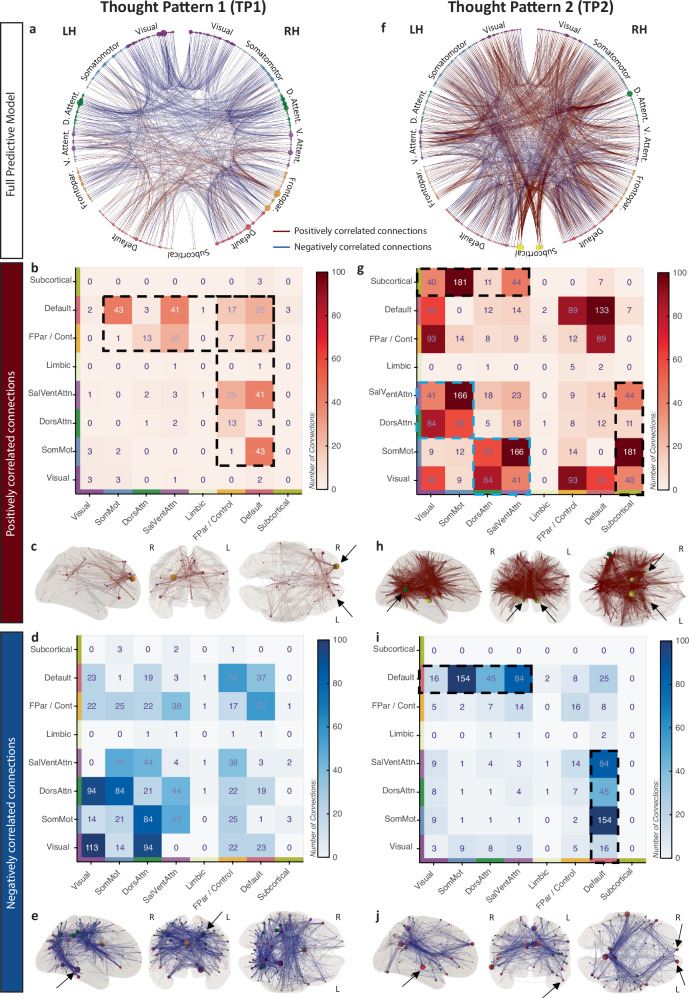
Subject-level cross-validation CPM (Connectome Predictive Modeling; *n* = 469 scans) Model for Thought Patterns 1 (TP1) and Though Pattern 2 (TP2). **a** Circos plot of the CPM model for the prediction of loadings on TP1 axis. Red lines and dark blue lines denote, respectively, connections that correlate positively or negatively with the prediction target. **b**, Counts of within- and across-network connections that correlate positively with TP1. **c**, Connections positively correlated with TP1 on a glass brain. **d** Counts of within- and across-network connections that correlate negatively with TP1. **e** Connections negatively correlated with TP1 on a glass brain. **f**–**j**, Equivalent results to **a**–**e** but using as the prediction target TP2 loadings instead of TP1. The color of circles representing ROIs in Circos plots and glass brains denote network membership, and the size of the circle the number of connections that include a given ROI (i.e., its degree). Source data are provided as a Source Data file.

Next, Fig. 7 shows the subject-level cross-validation CPM models for predicting factor scores on the two Thought Patterns previously described. The CPM model for TP1 (Fig. 7a–e) is sparser (915 connections | 1.3%) than the one for TP2 (1635 connections | 2.3%; Fig. 7f–j). For TP1, positively correlated connections involve primarily nodes of the Frontoparietal Control and Default Mode networks (black dashed rectangles in Fig. 7b), with highest degree nodes sitting in bilateral dorsolateral prefrontal cortex (black arrows in Fig. 7c). Conversely, connections that correlate negatively with TP1 tend to concentrate in central and posterior areas of the brain that belong primarily to the Visual and Dorsal Attention networks. Highest degree nodes for this second portion (negative correlations) of the model include the left medial intraparietal sulcus, part of the Dorsal Attention network, and left para-hippocampal area, which is part of the Visual network (black arrows in Fig. 7e).

For TP2, the CPM model has 1215 (1.7%) positively correlated connections widely distributed across all networks, except the Limbic network. That said, larger connection counts occur for connections between subcortical regions and regions from both attentional networks and primary unimodal networks (black dashed rectangle in Fig. 7g), as well as between both attentional networks and primary unimodal networks (cyan dashed rectangle in Fig. 7g). Similarly, top degree nodes include bilateral thalami and putamen, as well as the right temporo-parieto-occipital junction, which is part of the Dorsal Attention network (black arrows in Fig. 7h). Regarding the 420 (0.6%) connections that negatively correlate with TP2, these are primarily connections between nodes of the Default Mode network and nodes in both Attention and unimodal primary networks (black dashed rectangle in Fig. 7i). In this case, highest degree nodes are located primarily in the Default Mode network (black arrows in Fig. 7j).

**Fig. 8:**
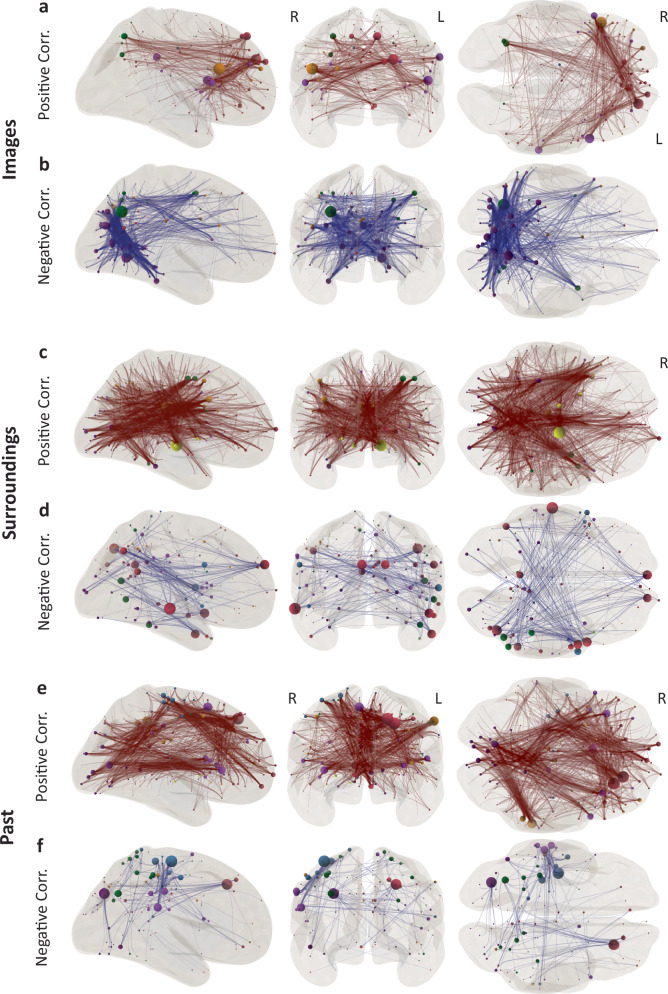
Subject-level cross-validation CPM (Connectome Predictive Modeling; *n* = 469 scans) models for individual items related to the form and content of thought that we could predict above significance. **a,** **b** Predictive model for Images. **c**, **d** Predictive model for Surroundings. **e**, **f** Predictive model for Past. In all instances, positively correlated connections are shown on the top row (**a**, **c**, **e**) in dark red, and negatively correlated connections on the bottom row (**b**, **d, f**) in dark blue. Node size denotes degree and node color denotes network membership (see Fig. 3a). Source data are provided as a Source Data file.

Figure 8 shows the subject-level cross-validation CPM models for the three individual questionnaire items about the form and content of thoughts that could be predicted significantly, namely: Images, Surroundings and Past. The CPM model for Images (Fig. 8a, b) shares features with that of TP1 (Fig. 7c, d), of which Images is a prominent characteristic (Fig. 1d). Common features between the Images and TP1 models include a tendency for positively correlated connections (dark red connections, Figs. 7c and 8a) to involve regions in dorsolateral prefrontal cortex and negatively correlated connections to concentrate on posterior aspects of the brain, primarily in occipital cortex (dark blue connections, Figs. 7e, 8b). Some level of agreement was also observed for the CPM model for Surroundings (Fig. 8c, d) and that of TP2 (Fig. 7h, j), of which Surroundings is the most prominent characteristic (Fig. 1e). In this instance, similarities across models include the heavy contribution of subcortical regions to positively correlated connections, and of Default Mode network nodes to their negatively correlated counterparts. For Past, which was not as strongly associated with either Thought Pattern, we observe a unique CPM model primarily composed of positively correlated edges that has its two higher degree nodes located in left frontal regions of the Default Mode network (Fig. 8e).

Finally, as in the NBS-based analyses, we examined whether untested correspondences between experiential measures and participants’ traits might have influenced the CPM results. To do this, we computed Pearson correlations between prediction targets (the 11 sNYCQ items and the 2 thought-pattern axes) and the 46 behavioral metrics listed in Supplementary Table 9. Fig,e 9 summarizes the results. Two sNYCQ items showed significant associations with behavioral traits: *Negative* correlated with six measures (BISBAS_BIS, HADS-D, IAT, PSSI_BL, PSSI_SU, and PSSI_DP), and *Intrusive* correlated with one (S-D-MW_delib). We also observed significant correlations between Thought Pattern 1 and HADS-D, and between Thought Pattern 2 and HADS-D, BISBAS_BIS, and PSSI-DP.

**Fig. 9:**
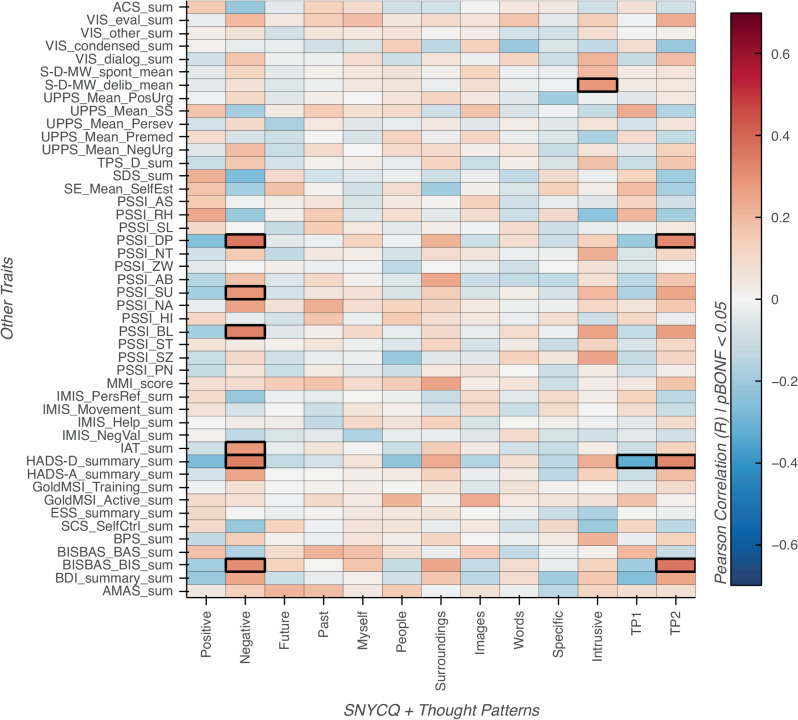
Correlation between subject-level cross-validation CPM prediction targets and other trait measures. Cells are colored according to the Pearson correlation. Relationships that survived p_Bonf_ < 0.05 (two-sided) are marked with a bold black outline. Abbreviations for traits showing significant correlations: BISBAS_BIS_sum Inhibition component of the Behavioral Inhibition and Approach System Summary Metric, HADS-D Depression Subscale of the Hospital Anxiety and Depression Scale, IAT Internet Addiction Test, PSSI_BL, Spontaneous Borderline subscale of the Personality Style and Disorder Inventory (PSSI), PSSI_SU,Self-critical/Insecure subscale of the PSSI; PSSI_DP Quiet/Depressed Subscale of the PSSI, S-D-MW_delib_mean Deliberate Mind-Wandering subscale of the Spontaneous and Deliberate Mind-Wandering Scale. All other abbreviations available in Supplementary Table 9. Source data and number of samples entering each test are provided as a Source Data file.

## Discussion

This investigation provides strong evidence for the key—yet often overlooked—role that trait-like aspects of the subjective in-scanner experience plays in resting-state FC. Our results demonstrate the enhanced research value of annotating rsfMRI data with experiential information to facilitate a more direct exploration of what aspects of intrinsic activity during rest are related to specific attributes of self-generated thoughts. Importantly, this study offers three unique contributions: 1) subjects have similar experiences across successive rsfMRI sessions, 2) systematic differences in in-scanner experiences yield inter-population differences in resting-state FC, 3) resting-state FC can predict some aspects of in-scanner experiences. These findings are relevant to those studying the neural correlates of spontaneous thought, and more generally, to those who use rsfMRI to map the functional connectome of healthy and diseased populations. We discuss each of these contributions and their implications below.

First, we show that for subjects with multiple rsfMRI scans in our sample, those scans are often grouped together when we divide the sample according to reported experience (Fig. 2). We also show that it is possible to successfully identify subjects based on their inner experience, although to a lower degree than what is possible based on FC (Fig. 2). While identification rate based on FC was almost perfect, identification rate based on experience was only 19%. That said, both identification rates were significant according to a permutation analysis. Differential identifiability analyses further confirm that sNYCQ responses within a subject are more like each other than across subjects. Although I_Diff_ was higher for experiential than FC data, we do not believe this indicates that in-scanner experience is “a better fingerprint” than FC (as clearly the identification rate analysis indicates otherwise). We believe this discrepancy between the identification rate and I_Diff_ is due to I_Diff_ only considering differences in mean values between I_Self_ and I_Other_, and ignoring the spread of distributions across these means. Recent work^53^ has demonstrated how this omission in the definition of I_Diff_ can lead to somehow contradictive results. When we plot the distributions of I_Self_ and I_Other_ values for experiential data (Fig. 2c) and FC data (Fig. 2e) we can see that indeed the separability of these distributions is much larger for the FC data, in agreement with the observations made in terms of the identifiability rate.

Despite the above-discussed discrepancies, the three analyses lead to the same conclusion: subjects tend to fall back on similar thought patterns—at least at the level of detail captured by the sNYCQ—every time they participate in a resting-state session. This agrees with prior literature on the topic^54–56^. For example, Diaz et al.^54^, using the Amsterdam Resting-State Questionnaire (ARSQ), found a high level of test-retest reproducibility for the seven dimensions of experience captured by that tool (Discontinuity of Mind, Theory of Mind, Self, Planning, Somatic Awareness, Comfort, and Sleepiness) across two resting-state scans that occurred at the beginning and end of a multi-task scanning session. In a follow-up study^56^, using an updated version of the ARSQ that also quantifies Health Concerns, Visual Thought and Verbal Thought, the authors found stable and significant test-retest reliability for intervals of 3–32 months in all explored dimensions of in-scanner experience except for Sleepiness and Health Concerns. These reports, combined with ours, suggest that subjects default back to overall similar thought patterns and feelings inside the scanner, suggesting that summary characteristics of in-scanner experience captured with retrospective tools should be considered trait-like, as opposed to state-like, characteristics, given their long-term temporal stability.

This finding has important interpretational and analytical implications. First, the high within-subject test-retest reliability of rsFC has often been used as an argument against the possibility of “resting cognition” being a contributing factor to rsFC under the assumption that “resting cognition” would vary from scan to scan^3,57^. While our results, as well as those of others discussed in the previous paragraph, do not imply that subjects have the same exact thoughts and feelings during different scan sessions, it demonstrates that overall high-level descriptors of in-scanner experience (such as those captured by sNYCQ items) might be more static and reproducible within-subject than originally assumed. In fact, this propensity of subjects to have similar thought patterns every time they enter the MRI scanner can explain why in-scanner experience being a modulatory factor of rsFC is compatible with rsFC having high test-retest within-subject reliability. Second, these results also imply that simple within-subject, across-scan averaging will not be efficient at removing in-scanner experiential effects, should that be desirable. An alternative option might be to collect experiential information using instruments such as the sNYCQ or the ARSQ^54,56^, so that they can be included in analytical models. However, their inclusion requires careful consideration. Treating experiential measures as nuisance covariates by default could remove meaningful variance when in-scanner experience is related to the phenotype or process under investigation, as may be the case for rumination in depression. Conversely, when experiential variability is not itself the target of interest, modeling these measures may help reduce inter-individual variability that could otherwise obscure other sources of rsFC variance, including variance associated with other experience-independent neural processes occurring during rest. Accordingly, experiential measures should be incorporated in a hypothesis-driven manner, rather than automatically regressed out.

Our second research question aimed to quantify whether in-scanner experience is associated with resting-state FC patterns, thereby posing a confound (or an additional explanatory factor) for studies that compare rsFC across populations–a ubiquitous approach when looking to uncover clinically relevant FC patterns. Our analyses show significant differences in rsFC between data from two sets of healthy controls (matched for head motion, age, gender, and wakefulness levels) that differ in terms of the content and form of their in-scanner spontaneous thoughts (Fig. 3b–d). Importantly, the spatial extent of such differences—as quantified by percentage of regions and connections—is equivalent to that of clinical studies based on the same analytical methods (Fig. 3e, f). This observation suggests that systematic inter-population differences in what subjects feel and think while being scanned can affect interpretation in population-contrasting studies. Yet, the severity and interpretational challenges will be study-specific as they depend on (1), what aspects of spontaneous thoughts/experience are different across contrasted populations (e.g., overall valence, form, attentional focus, etc.) and (2), the spatial overlap between the connections that are modulated by the study inclusion criteria (e.g., presence/absence of a given clinical condition) and those due to in-scanner experience.

Beyond wakefulness (which has been extensively discussed elsewhere^6,7,42^), one aspect of in-scanner experience that we also found to be key is whether subjects focus their attention on their surroundings. Significantly stronger connections for scans with high loadings on TP2 (i.e., Set A)—which is most strongly associated with the Surroundings item of the sNYCQ (Fig. 1e)—involve primarily nodes of the Salience/Ventral Attention Network, the Dorsal Attention Network and primary somatosensory networks (red dashed rectangle in Fig. 3d). This pattern of stronger FC among these networks is compatible with the well-established role that these networks and their interactions play in the reorientation of attention towards stimuli^58,59^, and in shaping the content of spontaneous thoughts^21,55^. For example, a recent psychological framework of spontaneous thought^60^ suggests that the content of mental states can be constrained in two ways: deliberatively by regions of the Control Network, or automatically by the salience of affective and sensory stimuli via the Ventral Attention or Salience networks. In agreement with this framework, the contrast “*Set A > Set B*” shows increased connectivity between the Ventral Attention Network and Somatosensory and Visual regions, suggesting these thoughts may be characterized by stronger sensory salience. Additionally, engagement of the Dorsal Attention Network is viewed as exerting control to help maintain attention over time towards features of the environment^59,60^. This could also explain the observed higher connectivity between the DAN, VAN and sensory networks for scans in *Set A*. Similarly, the observed right hemisphere lateralization of connections that survived the *“Set A > Set B”* contrast is consistent with the known right dominance of spatial attentional processes^61,62^. Such compatibility between our observations and well-established functional roles for the regions and networks involved further supports the claim that the differences in rsFC observed here are indeed due to differences in in-scanner experience across scan sets.

Finally, we show that it is possible to predict aspects of in-scanner experience—namely Wakefulness, Images, Surroundings, Past, TP1, and TP2—using rsFC matrices. This further supports the idea that what subjects feel and think during rsfMRI scans leaves a reliable and detectable imprint on rsFC, and agrees with prior reports showing that it is possible to predict aspects of mind wandering—such as its presence^63^,self-relevance^64^, valence^64^ and prevalence^11^ —on the basis of rsFC. When we compare our accuracy estimates to studies that use CPM to predict clinical, cognitive and personality phenotypes (Supplementary Tables 7 and 8) we observe that our six models—despite satisfying individual criteria for statistical significance, and five of them surviving FDR-based correction—fall along the lower end of the distributions of accuracies reported in the literature (Fig. 5d). This can be interpreted in at least two ways. It could be argued that this observation suggests that in-scanner experience has a weaker signature in rsFC than phenotypes such as the severity of clinical symptoms, a person’s cognitive abilities, or personality traits. Alternatively (or additionally), lower accuracy could be the consequence of the imprecision and limited scope of retrospective introspection instruments for capturing relevant aspects of in-scanner experience during minutes-long rsfMRI scans (see Gonzalez-Castillo et al^2^. for a detailed discussion). Discerning between these two interpretations requires additional rsfMRI data with more accurate and extensive experiential annotations. Those could be obtained by combining first-person reports with in-scanner recordings (e.g., pupil size, gaze, skin conductance, videography^65^) that also carry information about subjects’ state of mind (e.g., stress and vigilance levels, focus of attention, occurrence of basic emotions, etc.). With such additional data, researchers will be able to evaluate whether accuracy improves as higher quality experiential information becomes available; and perform external across-dataset validation of CPM models, which is the optimal way to evaluate the generality of these models whenever possible^66^.

That said, and as we observed in the contrast analysis (Question 2), topological properties of our CPM models are reconcilable with current models of brain function. For example, the model for Wakefulness (Fig. 6a), being the densest of all models is compatible with the large functional reconfiguration that accompanies descent into sleep^6^. Thus, this CPM model includes many subcortical-to-cortical connections that positively correlate with Wakefulness (Fig. 6b,c) and many cortical-to-cortical connections that correlate negatively with Wakefulness (Fig. 6d,e). This pattern agrees with sleep onset being associated with reduced subcortical-to-cortical connectivity and increased cortical-to-cortical connectivity^6,67^. Also, the CPM model for TP2 recapitulates (Fig. 7g) the involvement of both attention networks and primary somatosensory networks observed in the contrast analysis (Fig. 3b-d). Finally, one prominent characteristic of the Images and TP1 models is the many within-visual connections that correlate negatively with reported levels of visual imagery (Figs. 7d, e, 8b). Prior work has shown that within visual network connectivity decreases when subjects engage in continuous visuospatial attention tasks^68,69^ and during natural movie viewing^70^. While the role of visual areas in visual imagery remains controversial (traditional views advocate in favor^71^, but recent meta-analytic^72^ and lesion studies suggest otherwise^73^), our results suggest that extended periods of visual imagery have a similar segregating effect within the visual network during rest to that of attention towards visual stimuli during active tasks.

Our findings, together with prior work (summarized above), suggest that although retrospectively measured in-scanner experience shows meaningful day-to-day variability—as reflected in its relatively low identification rate—it nevertheless exhibits sufficient stability to be conceptualized as a trait-like characteristic capturing the characteristic mental state of an individual while being scanned. Moreover, prior work has reported relationships between the prevalence of in- and out-of- the scanner mind wandering^11^, as well as broad similarities of off-task thought patterns in a laboratory setting and in everyday life^74^. All this evidence raises an important question: to what extent might the population differences and predictive relationships we observe reflect not thought content per se but other, redundant trait-like tendencies? Because the Mind–Brain–Body dataset includes extensive surveys probing everyday traits like personality, habitual behaviors, and cognitive control phenotypes, we were able to evaluate this possibility directly in our sample.

With respect to population-level differences, only one everyday trait (out of 46) significantly differed across scan sets (Fig. 4). Regarding predictability, only three in-scanner experiential characteristics (out of the five that could be predicted) significantly correlated with specific everyday traits (Fig. 9). Although sparse, these associations indicate that in-scanner experiences share some variance with everyday tendencies (and observation previously reported by others^75^). This suggests the potential for bidirectional influences: everyday dispositions may shape what individuals think about in the scanner, and in turn, help explain some of our broader observations. It is equally plausible, however, that in-scanner thoughts mediate why functional connectivity (FC) patterns differ between populations with distinct dispositional tendencies, or why those traits can be predicted from FC in the first place.

If in-scanner experience and everyday traits are interlinked, what is gained by characterizing the former? To illustrate this, we consider depression research and a recent precision rsfMRI study by Lynch et al.^76^ This study found that the frontostriatal salience network is nearly doubled in size in many individuals with depression—a feature that is stable across visits and uncorrelated with symptom fluctuations. In contrast, visit-to-visit changes in striatal connectivity with anterior cingulate and anterior insular regions tracked fluctuations in anhedonia and anxiety, respectively. These latter observations were possible because each scan was paired with detailed assessments of five depressive symptom dimensions in the days preceding the visit. Together, these results demonstrate how precision rsfMRI can identify both a stable biomarker of depression (network size) and, when paired with successive clinical assessments, symptom-specific dynamic biomarkers (striatal connectivity changes).

In our work, we find that individuals with a greater propensity for negative in-scanner content also score higher on everyday measures of negative affect (HADS-D, PSSI_DP) and on scales reflecting heightened sensitivity to negative cues (e.g., BIS). This suggests not only that subclinical inclinations—which are present in our sample despite the exclusion of individuals with diagnosed neuropsychiatric conditions—can influence FC estimates, but also that an additional layer of insight might have been available in Lynch et al. had in-scanner experience been measured. Without knowing the predominant mental state of participants during each scan, researchers may inadvertently group together scans obtained during euthymia with those acquired during heightened anhedonia, anxiety, or other symptoms. Such “mixing” may help explain why they could not reproduce their findings regarding anhedonia when using a cross-sectional approach, and it highlights how overlooking in-scanner experience differences can introduce unnecessary variance. This variance not only reduces effect sizes but, in extreme cases, may obscure the identification of additional dynamic markers of disease.

Notably, we were able to predict individuals’ tendency to focus on their surroundings or to think about the past, even though these two in-scanner experiential traits showed no detectable relationship with any of the everyday traits available in our dataset. Also, we could not predict the sNYCQ item “*Intrusive*” despite its correlation to the everyday trait “*SD_MW_delib*”; although this trait was previously associated with anatomical and functional changes in regions of the Control and Default Mode Networks using the same dataset^77^. Furthermore, many of the observed population-level FC differences (for example, greater right-hemisphere lateralization for scans in Set A), as well as the edge composition of successful CPM models, can be interpreted in light of well-established functional roles of the implicated regions. Thus, although the sparse correlations with everyday traits may reflect the limited scope of the behavioral measures included in the Mind–Brain–Body dataset, the fact that the FC patterns are consistent with current neurofunctional interpretations—and that double dissociations can be observed (that is, the ability to predict sNYCQ items not associated with everyday traits and vice versa)—strongly suggests that these experiential states affect FC estimates beyond what would be expected from everyday traits alone. This, in turn, supports the idea that experiential data provide valuable additional information, beyond estimates of everyday traits, for understanding which aspects of spontaneous activity during rest correspond to specific dimensions of conscious experience.

Importantly, this does not imply (nor does it deny) that FC patterns might capture, on a volume-by-volume basis, the sequential unfolding of moment-to-moment thoughts, feelings, and sensations. This study rests on a similar assumption to most resting-state studies: data correlated or averaged over many minutes represents a neurally meaningful average brain state. This does not preclude neurally meaningful changes in brain states on shorter time scales that are also likely to exist. Yet, establishing such a link between fMRI and introspective states on such shorter time scales would require an entirely different methodological framework—one in which the dynamics of conscious experience are measured repeatedly within a scan^17,18,63,78^, or ideally, at a temporal resolution comparable to the fMRI recordings themselves. Such an approach is challenging: active probing during scanning inevitably disrupts the very processes we aim to observe (i.e., the spontaneous emergence of thoughts and mind wandering), and within-scan probes engage additional cognitive operations that may be confounded with the experiential processes of interest. A promising alternative may be to concurrently acquire other physiological signals known to covertly provide information about the content and form of conscious experience^79^ and combine those with a limited number of in-scanner probes, which would be less disruptive. Moreover, integrating first-person reports—whether in-the-moment or retrospective—with such physiological, covert indexes of consciousness may help mitigate some of the uncertainties inherent to introspective reports (e.g., presuppositions, biases, limited self-awareness)^12,80,81^. Future research should evaluate the feasibility of these approaches.

Finally, it is important to discuss why our findings are not incompatible with views suggesting that spontaneous activity also subserves biological purposes beyond sustaining conscious experience during rest. In a recent paper titled “Brain activity is not only for thinking”, Laumann and Snyder list seven scientific observations difficult to reconcile with the idea that all ongoing BOLD activity directly reflects cognition and behavior. Their arguments are valid, but they do not contradict our view that some, not all, spontaneous BOLD activity during awake rest reflects ongoing cognition and behavior, and that this cognitive contribution should be considered when interpreting resting-state data.

One scientific observation emphasized by Laumann & Snyder is that rsFC structure remains present and broadly similar—but far from identical—across states that differ in consciousness levels (awake, sleep, anesthesia) and cognitive demands (task vs. rest). While this would indeed challenge the idea that all spontaneous activity reflects cognition, it does not contradict the possibility that some spontaneous activity reflects cognition, and that during awake rest, this portion meaningfully contributes to rsFC estimates. Our across-set comparisons and connectome models—though widespread—are also limited: many connections do not show any detectable link to ongoing cognition, despite contributing nodes not being silent (i.e., presenting spontaneous activity). This is consistent with our claim that cognition accounts for part, but not all, of spontaneous fluctuations.

Another observation they highlight concerns the high consistency of networks across sessions. As we already discussed above, this can be reconciled with our view because resting-state cognition, as measured by retrospective summary metrics, shows trait-like properties and is somewhat consistent within-subject across repeated scanning sessions.

The final two arguments raised by Laumann & Snyder—that rsFC structure is consistent at the population level and that similar structures appear across mammalian species—are also not incompatible with our position. Regarding population-level consistency, the same critique would apply to processes such as learning consolidation or plasticity, whose specific contents likely vary across individuals and across time (e.g., what information is being consolidated). Our claim is not that specific thought content (e.g., thinking of a black cat vs. a white dog) produces large rsFC effects. Rather, we propose that higher-level patterns of in-scanner experience (e.g., a tendency to engage in internal conversation, ruminate, or feel anxious in the scanner) can partly explain reproducible features of rsFC. For Laumann & Snyder’s cross-species arguments, it is difficult to draw strong conclusions given the limited understanding of conscious experience—or its absence—in nonhuman mammals.

In summary, this work shows that what subjects think and feel during rsfMRI scans matters. It extends prior work showing how seemingly irrelevant differences in pre-scanning instructions (e.g., ‘let your mind wander’ vs. ‘think of nothing’^82^) can alter FC patterns as well as past studies demonstrating that in-scanner experience and spontaneous thought patterns can be related to resting-state signals^13,14,21,55,83–85^. It highlights how introspective reports can enhance the interpretation of rsfMRI by attaching a meaningful neurocognitive interpretation to findings of differing rsFC. Importantly, we do not claim that in-scanner experience and spontaneous thought patterns are the only (or primary) factor driving rsFC. As briefly mentioned in the introduction, other neurobiological and cognitive processes (e.g., memory consolidation, homeostatic processes, etc.) might be equally (or more) important. Similarly, other sources of meaningful inter-subject variability, such as individual differences in the spatial arrangement of brain regions—which has been shown to be a good predictor of behavioral and lifestyle phenotypes^86^ —must also be considered. Future research should elucidate the relative contributions of these and other factors. Similarly, our results use rsfMRI, but they also apply to proposed alternative experimental designs, such as naturalistic protocols. While naturalistic stimuli can induce synchronized neural responses across subjects (offering analytical approaches not available to rsfMRI^87^), subjects might still experience the same movie (or audio story) differently on repeated presentations (e.g., by focusing on different aspects, recalling prior presentations, or eliciting different emotions), and therefore accurate interpretation of naturalistic-based results might also ultimately require experiential annotations, as we propose for resting-state.

Resting-state fMRI occupies a central place in neuroimaging research, yet its inability to fulfill clinical standards (i.e., sufficient specificity and sensitivity) and often unsatisfactory interpretation have stalled progress in recent years. Here, we show that considering the role that in-scanner experience plays in rsFC might address these issues by accounting for unexplained variance in the data. This is not an insurmountable challenge. Much of that variance might be properly modeled once we learn what aspects of experience matter most, their co-occurrence with different clinical conditions, and how to accurately measure their presence/intensity during fMRI scans. Ultimately, this work suggests that it is possible to move beyond the current impasses if we, as a community, are willing to embrace and help resolve the many complexities that arise when incorporating first-person data into our research agendas.

## Methods

This work was conducted in compliance with a protocol approved by the Institutional Review Board of the National Institute of Mental Health in Bethesda, MD, USA. Regarding data acquisition, and according to Mendes et al.^35^ (the Scientific Data publication that accompanied the public release of the dataset used here), all participants provided written informed consent (including agreement to their data being shared anonymously) prior to their participation in the study. Participants received monetary compensation for their participation. The study protocol under which the data were originally acquired was approved by the ethics committee at the medical faculty of the University of Leipzig (097/15-ff).

### Dataset

This work is conducted using portions of the publicly available Max Planck Institute Leipzig Mind-Brain-Body dataset^35^. This dataset includes, among other items, resting-state scans (15.5 mins long, 2.3×2.3×2.3 mm, TR = 1.4 s, 3 T) annotated with retrospective self-reports of information regarding the content and form of thoughts participants had during the scan (see Table 1). For each resting-state scan, it also contains information about participant’s subjective perception of how awake they remained during the scan, as well as age range (in 5-year intervals) and gender. All information about scanning parameters, demographics, and behavioral tests included in this dataset can be found in the Scientific Data publication that accompanies the release of the dataset^35^.

The original dataset contains a total of 788 resting-state scans acquired on 194 participants. The number of scans per participant varies between one and four. Scans are distributed across a maximum of two different scanning occasions. Only 693 scans (distributed across 175 participants; sex distribution: 90 males/85 females; 57 participants between 20-25 years, 67 between 25–30 years, 14 between 30–35 years, 13 between 35–55 years, and 24 between 55–80 years) had complete annotations about the form and contents of thoughts, as well as subjective reports of wakefulness. It is those 693 that we selected for our analyses.

### fMRI Data Pre-processing

For the 175 participants who had at least one resting-state scan annotated with in-scanner experience data, we attempted preprocessing of their T1 scans using the anatomical pre-processing pipeline distributed with the dataset (https://github.com/NeuroanatomyAndConnectivity). This pipeline includes the following operations: removal of T1-weighted image background (CBS Tools), cortical surface reconstruction (Freesurfer), and registration towards the MNI152 1 mm template (ANTs). For 25 subjects, the pipeline did not finalize successfully (e.g., errors in tissue segmentation, skull stripping, etc.). Resting-state scans from these subjects (96 scans) were not considered in the rest of this work (Supplementary Table 4).

Pre-processing of the remaining 597 resting-state scans proceeded with two steps. First, we used the initial portion of the functional pre-processing pipelines distributed with the MPI dataset to perform the following operations: discard the initial five volumes, head motion correction, spatial distortion correction, and co-registration to MNI space. Next, we performed the following three additional steps using AFNI^88^: temporal scaling by dividing by the mean, nuisance regression (motion, first derivative of motion, Legendre polynomials up to third degree, physiological noise regressors computed with the aCompCor method^89^), and temporal filtering [0.01–0.15 Hz]. Functional pre-processing failed for 13 resting-state scans due to missing functional or field map data (Supplementary Table 4).

For scans that completed the functional pre-processing pipeline, we used traces of relative displacement to mark as invalid volumes with a relative displacement above 0.3 mm. For any such volume, we also marked as invalid the preceding volume and the following two. Any scan with 30% or more of volumes marked as invalid was discarded from any additional analyses. This resulted in the removal of an additional 113 scans. Supplementary Table 4 summarizes the number of scans rejected at every pre-processing step.

### In-scanner experience reports analyses

#### Overview

Upon completion of each resting-state scan, subjects completed the *sNYCQ* (short New York Cognition Questionnaire), a 12-question survey that gathers information about different aspects of the form and content of thoughts incurred during the immediately preceding resting-state scan. Because the *sNYCQ* is collected immediately after each run and retrospectively queries the preceding measurement period, it provides a summary-level characterization of in-scanner experience rather than a temporally resolved account of within-run experiential fluctuations.

In-scanner experience data were divided into two parts: one contained responses to the 11 questions about the form and content of in-scanner thoughts, and the second was a vector with the response to the wakefulness item (Fig. 1a). The dimensionality reduction and clustering steps described below were conducted using only the first part (i.e., answers to all questions except the one about wakefulness). Answers to the wakefulness item were only used to evaluate potential differences in wakefulness levels across scan sets generated via clustering and as a target for prediction (see Connectome Predictive Modeling section below).

#### Data scaling

Answers to all questions, except wakefulness, were scaled using scikit-learn *RobustScaler*. This scaler removes the median and scales the data by the inter-quantile range. This form of scaling was performed to avoid the excessive influence of outliers in the scaling process.

#### Outlier detection

Outlier detection was based on the squared Mahalanobis distance of each scan to the sample’s mean. We marked as outliers those scans with a Mahalanobis distance in the top 3% quantile. This resulted in the elimination of 2 scans.

#### Clustering

To separate scans into two sets with differentiated inner-experience, we decided to apply two different clustering algorithms to the in-scanner experience data (11 questions) following robust scaling. To be clear, clustering was performed in the original 11D space, not the low-dimensional space generated by T-SNE or ICQF. Working with two different clustering methods allows us to evaluate the robustness of results against the clustering technique.

The two selected methods were K-Means^90^ and Gaussian Mixture Modeling^91^; both as implemented in the Python library scikit-learn^92^. K-Means was chosen as a representative hard-clustering method often used in the neuroimaging literature. Gaussian Mixture Modeling (GMM) was chosen because of its soft-clustering nature (i.e., it provides membership probabilities) and because it allows non-spherical clusters. In both instances, we set k = 2.

The consistency of clusters across methods was evaluated with the Adjusted Rand Index^93^ (ARI). Next, the ability of each method to capture true structure in the data was evaluated using the Silhouette Index^94^ (SI). Finally, for the method with the best SI, we then performed a bootstrapping analysis (N = 1000; subsamples size=80%) to evaluate cluster stability under random resampling.

Scans were initially given one of three labels according to the outcomes of the GMM algorithm: Set A, Set B, or Ambiguous. Scans were assigned a group label (A or B) if the probability of belonging to one of such groups was greater than 0.8. Otherwise, the scan was given the Ambiguous label.

To evaluate what aspects of in-scanner experience drive the cluster separation, we computed Cohen’s d across sets for each of the 12 original items in the SNYC questionnaire. We also performed a Mann-Whitney U test on each item to check for statistical significance.

#### Dimensionality reduction

To explore the structure of in-scanner experience reports, we first computed the Pearson’s correlation between the 11 in-scanner experience items. This revealed the presence of some relationships between sNYCQ items (Fig. 1b). Next, we created two low-dimensional representations of the data: one using Interpretability Constrained Questionnaire Factorization (ICQF)^43^, and a second one using T-distributed Stochastic Neighbor Embedding^95^.

#### Dimensionality reduction with ICQF

Answers regarding the form and content of in-scanner thoughts (matrix *M*_469 scans X 11 questions_; Supplementary Fig. 5a) and demographic information (matrix *C*_469 scans X 5 demographic encoding variables_; Supplementary Fig. 5c) were input to the Interpretability Constrained Questionnaire Factorization (ICQF)^43^ algorithm to generate a low-dimensional representation of the data. We chose this matrix decomposition technique as one of our dimensionality reduction methods, as opposed to Principal Component Analysis, because it is better suited for distributions that are both non-negative and non-Gaussian (that was the case for these data; Supplementary Fig. 1), ensure positive loadings in the low dimensional space (which is key for interpretational purposes), and account for potential confounding effects between demographics and questionnaire items.

In a nutshell, the ICQF algorithm attempts the following matrix decomposition (Supplementary Fig. 5):

1 $${{\bf{M}}}=\,\left[{{\bf{W}}},{{\bf{C}}}\right]\bullet {\left[{{\bf{Q}}},{{{\bf{Q}}}}_{{{\bf{c}}}}\right]}^{{\prime} }$$

where M and C are the inputs to the algorithm described above. W is the resulting low-dimensional representation (*d* = number of dimensions) of the data (469 scans X *d* Dimensions) with entries constrained to the range [0,1]. Q is a 11 questions X *d* Dimensions matrix with values constrained to the range [0,100] and provides information about how each dimension in the lower space relates to the original 11 questionnaire items. ${Q}_{c}$ is an 11-question X 5 demographic items matrix with values also in the range [0,100] that contains information about how age and gender relate to the original questionnaire items. Finally, as stated above, C is a 469 ×5 matrix with demographic information encoded as follows: the first column models an intercept (all ones), the next two columns encode age range using hashing encoding constrained to the [0,1] range, and the last two columns encode gender using one-hot encoding. In Eq. 1 above, $\left[A,B\right]$ denotes horizontal concatenation of matrices A and B.

Given M and C, the ICQF algorithm finds Q, ${Q}_{c}$ and W in a data-driven manner (see Lam et al^43^ for additional details). The ICQF algorithm has three hyperparameters, namely the number of dimensions (d), the sparsity level for the W matrix (${\beta }_{W}$), and the sparsity level for the Q matrix (${\beta }_{Q}$). We used cross-validation to estimate (in a data-driven manner), the optimal values for these three parameters. The range of values explored for each hyper-parameter and optimal values selected are reported in Supplementary Table 5.

#### Dimensionality reduction: T-SNE

Scaled answers to the sNYCQ were used as input to the T-SNE algorithm. Two key hyperparameters of the T-SNE algorithm are dimensionality and perplexity. We relied on trustworthiness^96^ —an estimate of how well a given embedding preserves local distances—to select these two hyper-parameters in a data-driven manner. The explored hyper-parameter space was: dimensionality = {1,2,3}, perplexity = {5,9,10,13,15,18,20,30,40,50}. The values with the highest trustworthiness (0.94) were dimensionality = 3 and perplexity = 18.

To aid with the interpretation of how T-SNE dimensions relate to the original items in the sNYCQ survey, we modeled each of the sNYCQ items as a linear function of the three T-SNE coordinates. We then used these coefficients to draw biplot arrows (black and red lines in Fig. 1.k) depicting the directions of maximal change for each sNYCQ item in the 3D T-NSE embedding space.

#### Scan set membership consistency

For scans from subjects that were scanned at least 3 times (116 subjects/442 scans), we looked at the counts and probabilities of how often: (a) all scans from a given subject belong to the same scan set, (b) all scans but one from a given subject belong to the same scan set, (c) any other configuration of set membership.

#### Differential Identifiability

We estimated differential identifiability following methods previously described by Amico et al.^45^ For this part of the analyses, we considered all scans from subjects that were scanned at least twice (462 scans / 12.6 subjects). We first computed Pearson’s correlation between all scan pairs, with scans being defined in terms of the scaled answers to all sNYCQ items except vigilance. We then used this matrix to compute the following three statistics:

* Self Identifiability (I_Self_): average correlation for all within-subject scan pairs.

* Others Identifiability (I_Other_): average correlation for all across-subject scan pairs.

* Differential Identifiability (I_Diff_): $$100*\,\left({I}_{{Self}}-{I}_{{Other}}\right)$$

#### Identification rate

For each scan, we searched for the scan with the highest Pearson’s correlation between its descriptions in terms of the 11 sNYCQ items. This is equivalent to the procedures originally described by Finn et al.^31^ If both scans belong to the same subject, we marked that as a successful identification attempt; otherwise, as a failure. Identification rate is the ratio of successes relative to the total number of scans, multiplied by 100.

To assess the significance of observed identification rates, we performed a permutation analysis with 10,000 random permutations. In each permutation, we randomly shuffled subjects’ identities associated with scans and proceeded as described above.

### Additional outside-the-scanner behavioral surveys

The Mind-Brain-Body dataset also includes 31 surveys measuring trait-like behaviors from five different categories: personality/habituation behaviors (21 surveys), mind wandering/mindfulness (4 surveys), synesthesia (1 survey), cognitive control/sustain attention (1 survey), and creativity (4 surveys). These surveys were administered only once per subject, as opposed to the experiential surveys that were administered following each resting-state scan (as detailed above).

Here, we will use these behavioral surveys to evaluate if any of the multiple traits assessed by the available outside-the-scanner behavioral surveys can explain any of the observations we make regarding population differences and prediction accuracy based on FC data. As such, we constrain our investigation to summary survey metrics available for at least 70% of subjects entering this population and predictability FC-based analyses. Supplementary Table 2 lists the 19 behavioral surveys that passed our selection criteria. This table also includes the number of summary metrics available with each survey (which adds to a total of 46 metrics) and a brief description. Supplementary Table 9 lists these 46 metrics. Additional information can be found on the Scientific Data publication^35^ that accompanies the release of the Mind-Brain-Body Dataset.

### Role of external traits in population differences

To assess whether traits beyond in-scanner experience might account for the observed FC differences between sample subsets (e.g., Sets A and B), we examined whether any of the summary metrics listed in Supplementary Table 9 varied systematically across sets. To do so, we performed two-sided independent T-tests and Mann–Whitney tests on each metric, comparing the distributions of values for participants who appeared exclusively in one set or the other.

### Role of external traits in predictive analyses

Similarly, to assess the potential influence of these traits on the prediction analyses described below in the subsection *“Functional Connectivity Analyses – Connectome-Based Predictive Modeling”*, we computed Pearson correlations between the 46 behavioral metrics listed in Supplementary Table 9 and all in-scanner experience descriptors being predicted (i.e., the 11 SNYCQ items related to the form and content of thoughts and the two Thought Pattern latent axes identified using ICQF). Because behavioral traits were assessed once per participant, whereas experiential measures were available for every scan, experiential data were averaged within participants across all scans prior to computing correlations. In other words, in these analyses, each participant’s in-scanner experience was represented by the mean of their reported experiences across all available resting-state scans.

### Functional connectivity analyses - statistical differences across scan sets

#### Parcellation

Following pre-processing, we computed representative time-series (spatial mean across voxels) and FC matrices separately for each scan using a modified version of the 400ROI/7 Networks Schaefer atlas^46^ using the AFNI^88^ program *3dNetCorr*. Thirteen ROIs were removed from the atlas prior to generating the FC matrices because they include regions suffering from excessive signal dropout. Those correspond to the 12 regions forming the Limbic network and one region part of the Default Mode network labeled as “*LH_Default_Temp_2*”. Additionally, 8 subcortical regions for the AAL atlas^47^ (bilateral thalami, putamen, striatum, and pallidum) were added. Supplementary Fig. 3 shows the final version of the atlas used in the analyses.

#### Network-based-statistics: significant testing

We used the Network-Based Statistic (NBS^48^) procedure to identify significant differences in FC between scan sets on opposite ends of the in-scanner experience space defined above. The NBS algorithm proceeds as follows. First, the procedure identifies individual edges that surpass an edge-level significance level of *p* < 0.001 using a two-sample T-test on a design matrix (Supplementary Fig. 6) that encoded both set membership (e.g., *Set A* or *Set B*) and subject identity, to account for the fact that there were repeated scans per subject in the sample. Next, the algorithm looks for connected components of supra-threshold edges and computes their extent (i.e., size). This is a network equivalent of how cluster size is computed on supra-threshold statistical activity maps. Third, a null distribution of connected component sizes is generated using 5000 permutations of scan labels. This null distribution is used to evaluate the statistical significance of observed connected sets of supra-threshold edges. Only those with a component-level *p* < 0.05 (i.e., p_NBS_ < 0.05) are rendered significant and retained for interpretation, which is equivalent to family-wise error corrected significance for the identified network components.

#### Laterality Index

To quantitatively evaluate if connections involve primarily regions from one hemisphere over the other, we computed the FC Laterality Index (${{FC}}_{{laterality\_index}}$) using the following formula:

2 $${{{\rm{FC}}}}_{{{\rm{laterality}}}\_{{\rm{index}}}}=\,\frac{{{\rm{LL}}}-{{\rm{RR}}}}{{{\rm{LL}}}+{{\rm{RR}}}}$$

where *LL* is the number of left intra-hemispheric significant connections and *RR* is the number of right intra-hemispheric significant connections. The ${{FC}}_{{laterality\_index}}$ ranges between −1 (right-dominant) to 1 (left-dominant).

This formula was adapted from its equivalent standard formulation for activity-based results^97,98^ by substituting active-voxel counts by number of significant connections.

### Functional connectivity analyses – connectome-based predictive modeling

We used connectome-based predictive modeling^31,51^ (CPM) to predict individual responses to all items of the experiential questionnaire, as well as the loadings on both axes of the low-dimensional representation of in-scanner thoughts (i.e., TP1 and TP2). To mitigate the effect of head motion confounds, we residualized target variables (i.e., image score, TP1) with respect to head motion before training the models.

CPM is a technique that builds predictions of behavioral or clinical scores from whole-brain FC data. A detailed description of the method can be found in Shen et al.^51^. Here, we will briefly describe the specifics of our implementation, which matches that of Finn & Bandettini^99^. First, data is divided into training and test sets using a 10-fold cross-validation procedure. Second, in each training set, we looked for edges that significantly correlated (*p* < 0.01), positively or negatively, with the target of prediction. Next, those edges were used to construct the following model:

3 $${{{\rm{y}}}}_{{{\rm{pred}}}}={{{\rm{\beta }}}}_{0}+{{{\rm{\beta }}}}_{1}\left({{{\rm{X}}}}_{{{\rm{pos}}}}\right)+{{{\rm{\beta }}}}_{2}\left({{{\rm{X}}}}_{{{\rm{neg}}}}\right)+\,{{\rm{\epsilon }}}$$

where ${X}_{{pos}}$ and ${X}_{{neg}}$ denote the sum of connectivity strength for all edges that correlated positively and negatively, respectively, with the prediction target. Finally, we used this model on the testing set to predict the target metric. These steps were conducted over the 10 different folds to gather predictions for all scans.

Once those were available, we evaluated prediction performance in terms of Pearson’s correlation between observed and predicted values. It is worth noting that Pearson’s correlation is a relative indicator of predictive performance that affords us an understanding of how the model can distinguish between higher scores and lower scores. We emphasize that the results we report should be interpreted in this context, as it is possible that models with the same correlation between observed and predicted values can have varying levels of absolute error (i.e., mean squared error).

We repeated this whole process 100 times to ensure our results were robust to variability in how the data was split into training and testing folds during the cross-validation step. Given that our dataset includes multiple scans per subject, it was important to consider subject identity during the split of the data into training and testing sets. For the primary analyses, when building folds, we ensured that all scans from the same subject were always included in the testing or training sets, but not across both. In other words, we did not use information from scans of one subject to predict a behavioral score for a different scan from the same subject. We refer to these results as subject-level cross-validation CPMs throughout the manuscript.

Additionally, and as secondary analyses, we also report results using a scan-level cross-validation scheme in which subject identity is not considered when building cross-validation sets. In this case, scans from the same subject could appear in both sets. Results from these secondary CPM analyses are only reported as supplementary materials to emphasize the importance of considering repeated measures when prediction targets behave primarily as traits.

#### Statistical testing

To assess the statistical significance of the predictions, we generated a null distribution by running the whole CPM pipeline using randomized labels 10,000 times. We then computed a non-parametric *p*-value for the observed model accuracy using this formula^99^:

4 $$p=\frac{{{\rm{sum}}}\left({{{\rm{r}}}}_{{{\rm{null}}}} > {{\rm{median}}}\left({{{\rm{r}}}}_{{{\rm{obs}}}}\right)\right)+1}{{{{\rm{n}}}}_{{{\rm{null}}}}+1}$$

where ${r}_{{null}}$ represents prediction accuracy values across all 10,000 null permutations, ${{median}}(r_{{obs}})$ is the median prediction accuracy across the 100 attempts made on a given target and ${n}_{{null}}$ is the number of random permutations available (i.e., 10,000).

#### Predictive model visualization

In addition to the predicted scores, CPM also produces a network model of connections contributing to the prediction, both in the positive direction (i.e., positive correlation between edge connectivity strength and predicted score) and the negative direction (i.e., negative correlation between edge connectivity strength and predicted score). To generate these final models, we first calculated the average number of times a given edge contributed to a model across each of the 10 folds in each of the 100 iterations. We then only plotted and interpreted edges that were consistently included in the predictive models 90% of the time or more^99^.

### Literature search

#### Network-based statistics studies

Clinical studies that rely on Network-based Statistics (NBS) to search for significant differences in FC connectivity between clinical and healthy populations were identified as follows. We first used Scopus to find all open-access manuscripts that reference the original NBS work by Zalesky et al.^48^ and include the terms “functional MRI” (or variants) and “resting-state” (or variants) in the title, abstract or keyword list. The actual *Scopus* (http://scopus.com*)* query was:

*((REF(network-based AND statistic: AND identifying AND differences AND in AND brain AND networks) AND TITLE-ABS-KEY((“functional MRI” OR “fMRI” OR “functional magnetic resonance imaging”) AND (“rest” OR “resting-state” OR “resting state”))) AND PUBYEAR* > *2012 AND PUBYEAR* < *2024 AND (LIMIT-TO (DOCTYPE,“ar”)) AND (LIMIT-TO (LANGUAGE,“English”)) AND (LIMIT-TO (OA,“all”)))*

This query returned a list of 402 documents. Next, going in reverse chronological order (oldest to newest), we search for the first 50 studies that report significant differences in resting-state static functional connectivity between healthy controls and a clinical population of interest based on some form of whole-brain parcellation and that were conducted on human subjects. These criteria, set to match our study's conceptual setup as much as possible, allowed us to discard search results with different study designs, such as pre-clinical studies, those based on task data, or dynamic aspects of connectivity, among many others.

For all 50 selected studies, we gathered—whenever available—basic information regarding sample size, scanning protocol (scanner strength, scan duration, repetition time), NBS hyperparameters (edge-level threshold, number of permutations, network-level threshold), parcellation size, and number and percentage of connections (and edges) found to be significantly different. For studies that report multiple contrasts (e.g., more than one clinical condition or multi-site studies) we gathered information for all available contrasts. When results are reported at multiple edge-level thresholds, we gathered results for the threshold more similar to ours (i.e., T > 3.1). Supplementary Table 6 shows the studies that matched our criteria.

#### Connectome-based predictive modeling studies

Studies that apply connectome predictive modeling to resting-state fMRI data were identified as follows. We first used Scopus to find all open-access manuscripts that describe the use of connectome predictive modeling to resting-state data in the abstract and cite the original work of Shen et al.^51^ describing the CPM method, using the following Scopus query:

*(TITLE-ABS-KEY ((“resting-state” OR “resting state”) AND (“connectome predictive modeling” OR “connectome based predictive modeling”) AND NOT dynamic AND NOT eeg AND NOT meg AND NOT fnirs) AND REF (using AND connectome-based AND predictive AND modeling AND to AND predict AND individual AND behavior AND from AND brain AND connectivity))*

This query returned a list of 88 documents. We then excluded from further analysis those studies that do not apply CPM to full brain ROI-based FC matrices (10 papers), use substantially modified versions of CPM (e.g., substitute OLS by SVR, have additional feature selection procedures, etc. | 8 papers), apply CPM only to task data (6 papers), report accuracy in a manner other than correlation between observed and predicted values (2 papers), report results for non-human data (1 paper), are based on non-fMRI data (1 paper), or are review manuscripts (3 papers). The remaining 57 studies reported accuracy for a total of 159 different CPM models, as several studies attempted prediction of more than one target and/or reported accuracy separately for the positive, negative, and combined CPM models. For studies that reported accuracy based on more than one internal cross-validation scheme, we report that for 10-Fold cross validation whenever possible (as this is the one used here). Each model was then assigned a label based on the nature of the prediction target: Clinical (53 models), Cognition (32 models), Personality/Well-being (26 models).

Of the 159 CPM models selected and labeled, only 91 report accuracy in terms of Pearson’s Correlation between observed and predicted values. It is these models that we use when constructing Fig. 3d. The remaining models, which used Spearman’s correlation, were discarded when generating the figure.

Supplementary Table 7 provides the list of 57 studies that passed our exclusion criteria. We report title, year, parcellation, number of regions of interest, cross-validation approach, and model accuracies. Supplementary Table 8 lists the studies found in Scopus that were excluded, as well as the exclusion criteria that applied to each particular study.

### Reporting summary

Further information on research design is available in the Nature Portfolio Reporting Summary linked to this article.

## Supplementary information

Supplementary Information

Reporting Summary

Transparent Peer Review file

## Source data

Source data

## Data Availability

This study is based on publicly available data as described in Mendes et al^35^ Imaging data can be accessed at ftp://ftp.gwdg.de/pub/misc/MPILeipzig_Mind-Brain-Body. Behavioral data is available at https://dataverse.harvard.edu/dataset.xhtml?persistentId=doi:10.7910/DVN/VMJ6NV. Additional access locations can be found in the Mendes et al. publication. In addition, Source data are provided with this paper.

## Code availability

Code used in this study is available at https://github.com/nimh-sfim/fc_introspection (release tagged as v4.0.0).
