# Supplementary material for: In-scanner thoughts contribute to resting-state functional connectivity
Source: Nat Commun. 2026 Jun 27;17:8058. doi: 10.1038/s41467-026-74953-6 (PMC13454257; doi:10.1038/s41467-026-74953-6)
Supplement: Supplementary file 2 [file 41467_2026_74953_MOESM2_ESM.pdf]

nature portfolio

Corresponding author(s): Javier Gonzalez-Castillo

Last updated by author(s): Apr 29, 2026

Reporting Summary

Nature Portfolio wishes to improve the reproducibility of the work that we publish. This form provides structure for consistency and transparency in reporting. For further information on Nature Portfolio policies, see our Editorial Policies and the Editorial Policy Checklist.

Statistics

For all statistical analyses, confirm that the following items are present in the figure legend, table legend, main text, or Methods section.

n/a	Confirmed	
☐	☒	The exact sample size (*n*) for each experimental group/condition, given as a discrete number and unit of measurement
☐	☒	A statement on whether measurements were taken from distinct samples or whether the same sample was measured repeatedly
☐	☒	The statistical test(s) used AND whether they are one- or two-sided<br>*Only common tests should be described solely by name; describe more complex techniques in the Methods section.*
☐	☒	A description of all covariates tested
☐	☒	A description of any assumptions or corrections, such as tests of normality and adjustment for multiple comparisons
☐	☒	A full description of the statistical parameters including central tendency (e.g. means) or other basic estimates (e.g. regression coefficient) AND variation (e.g. standard deviation) or associated estimates of uncertainty (e.g. confidence intervals)
☐	☒	For null hypothesis testing, the test statistic (e.g. *F*, *t*, *r*) with confidence intervals, effect sizes, degrees of freedom and *P* value noted<br>*Give P values as exact values whenever suitable.*
☒	☐	For Bayesian analysis, information on the choice of priors and Markov chain Monte Carlo settings
☒	☐	For hierarchical and complex designs, identification of the appropriate level for tests and full reporting of outcomes
☐	☒	Estimates of effect sizes (e.g. Cohen's *d*, Pearson's *r*), indicating how they were calculated

Our web collection on statistics for biologists contains articles on many of the points above.

Software and code

Policy information about availability of computer code

Data collection	This study uses a portion of a publicly available dataset, which has been fully described in the following publication: Mendes et al. "A functional connectome phenotyping dataset including cognitive state and personality measures" Sci. Data 6, 180307 (2009).

Data analysis	The code used for the analyses presented in this work is publicly available in Github (https://github.com/nimh-sfim/fc_introspection). The Github repository includes YAML files listing versions of all Python packages used for these analyses.<br><br>Pre-processing of anatomical images was conducted with the anatomical pre-processing pipeline distributed with the dataset (https://github.com/NeuroanatomyAndConnectivity). This anatomical pre-processing pipelines relies on the following software: FSL (v 5.0.10) Freesurfer (v 5.3.0), ANTs (v 2.2.0), MIPAV (v 5.4.2), and CBS Tools (v 3.0.8).<br><br>Pre-processing of fMRI data proceeded in two steps. First, we used the initial portion of the functional pre-processing pipelines distributed with the MPI dataset (also available at https://github.com/NeuroanatomyAndConnectivity) to perform the following operations: discard initial five volumes, head motion correction, spatial distortion correction, co-registration to MNI space. Next, we performed the three additional steps (temporal scaling, nuisance regression and temporal filtering) using the AFNI software.<br><br>Statistical analysis on functional connectivity matrices were performed using the MATLAB (R2023a) Network Based Statistics Toolbox (NBS, v 1.2). Connectome predictive modeling was implemented in Python (code available as part of the github repository that accompanies this publication). Visualization of connectivity models on glass brains generated with the MATLAB (R2023a) CONN software (v 22a). All other visualizations generated with seaborn, matplotlib, and hvplot Python libraries.

For manuscripts utilizing custom algorithms or software that are central to the research but not yet described in published literature, software must be made available to editors and reviewers. We strongly encourage code deposition in a community repository (e.g. GitHub). See the Nature Portfolio guidelines for submitting code & software for further information.

Data

Policy information about availability of data

All manuscripts must include a data availability statement. This statement should provide the following information, where applicable:

- Accession codes, unique identifiers, or web links for publicly available datasets
- A description of any restrictions on data availability
- For clinical datasets or third party data, please ensure that the statement adheres to our policy

This study is based on publicly available data as described in Mendes et al. "A functional connectome phenotyping dataset including cognitive state and personality measures" Sci. Data 6, 180307 (2009).

Imaging data can be accessed at ftp://ftp.gwdg.de/pub/misc/MPILeipzig_Mind-Brain-Body.

Behavioral data is available at https://dataverse.harvard.edu/dataset.xhtml?persistentId=doi:10.7910/DVN/VMJ6NV.

Additional access locations can be found on the Mendes et al. publication.

Research involving human participants, their data, or biological material

Policy information about studies with human participants or human data. See also policy information about sex, gender (identity/presentation), and sexual orientation and race, ethnicity and racism.

Reporting on sex and gender	Sex/gender were not considered in our study design. We report distribution of sex/gender across scan sets entering the Network-based Statistic Analyses.<br><br>The sample used in this study contains data from 90 males participants and 85 female participants.
Reporting on race, ethnicity, or other socially relevant groupings	We did not include analyses on race, ethnicity or other socially relevant grouping.
Population characteristics	Only age-group and sex/gender were available. As with sex/gender, distribution of age-group for scans entering the Network-based Statistics Analyses are reported.<br><br>The age distribution of the sample is: 57 participants between 20-25 years, 67 between 25-30 years, 14 between 30-35 years, 13 between 35-55 years, and 24 bewteen 55-80 years.
Recruitment	Please see "Recruitment and Inclusion Criteria" section of Mendes et al. "A functional connectome phenotyping dataset including cognitive state and personality measures" Sci. Data 6, 180307 (2009), as this manuscript describes in detail the publicly available dataset used int this work.
Ethics oversight	According to Mendes et al. "A functional connectome phenotyping dataset including cognitive state and personality measures" Sci. Data 6, 180307 (2009), data collection was approved by the ethics committee at the medical faculty of the University of Leipzig (097/15-ff)

Note that full information on the approval of the study protocol must also be provided in the manuscript.

Field-specific reporting

Please select the one below that is the best fit for your research. If you are not sure, read the appropriate sections before making your selection.

- [ ] Life sciences
- [x] Behavioural & social sciences
- [ ] Ecological, evolutionary & environmental sciences

For a reference copy of the document with all sections, see nature.com/documents/nr-reporting-summary-flat.pdf

Behavioural & social sciences study design

All studies must disclose on these points even when the disclosure is negative.

Study description	This is a quantitative study that jointly analyzed resting-state fMRI data annotated with experiental reports about the form and content of thoughts during the each resting-state scan. One such survey was completed for each individual scan.
Research sample	This study uses a portion of the publicly available Max Planck Institute Leipzig Mind-Brain-Body dataset. For this study we only used anatomical and experientally annotated resting-state fMRI scans. The original dataset contains a total of 788 resting-state scans acquired on 194 participants. The number of scans per participant varies between one and four. Scans are distributed across a maximum of two different scanning occasions. Only 693 scans(distributed across 175 participants) had complete annotations about the form and contents of thoughts, as well as subjective reports of wakefulness. It is those 693 that we selected for our analyses. Please see Table 3 for additional details of which scans entered final portions of the analyses. Additional details about the original sample can be found on Mendes et al. "A functional connectome phenotyping dataset including cognitive state and personality measures" Sci. Data 6, 180307 (2009).
Sampling strategy	Details about original sample selection can be found in Mendes et al. For this work we selected only scans that had experiential annotations available, and that completed all pre-processing steps successfully. Additionally, any scan with 30% or more of volumes marked as invalid (i.e., relative framewise displacement > 0.3mm) was discarded. Two additional scans were discarded because their associated experiential data was deemed outliers. This resulted in a final sample of 471 scans. Manuscript Table 5 summarizes the number of scans rejected at every pre-processing step.
Data collection	Details about data collection for the publicly available dataset used here can be found in Mendes et al. "A functional connectome phenotyping dataset including cognitive state and personality measures" Sci. Data 6, 180307 (2009).
Timing	The version of the data used in this work was made publicly available in 2020-07-22. Information about original data acquisition dates is not available.
Data exclusions	As mentioned above of the original 788 resting-state scans in the MPI dataset, we only used 469 These corresponds to scans that successfully completed all pre-processing, had experiential annotations not deemed outliers and met head motion criteria as stated in "Research Sample" above and described in Table 3 of the manuscript.
Non-participation	Unknown
Randomization	For the purpose of looking for establishing statistical differences in the network-based statistics portion of the analyses we used 5,000 random permutations. For the purpose of establishing significance for prediction accuracy of CPM models we used 10,000 null permutations.

Reporting for specific materials, systems and methods

We require information from authors about some types of materials, experimental systems and methods used in many studies. Here, indicate whether each material, system or method listed is relevant to your study. If you are not sure if a list item applies to your research, read the appropriate section before selecting a response.

Materials & experimental systems

n/a	Involved in the study	
☒	☐	Antibodies
☒	☐	Eukaryotic cell lines
☒	☐	Palaeontology and archaeology
☒	☐	Animals and other organisms
☒	☐	Clinical data
☒	☐	Dual use research of concern
☒	☐	Plants

Methods

n/a	Involved in the study	
☒	☐	ChIP-seq
☒	☐	Flow cytometry
☐	☒	MRI-based neuroimaging

Plants

Seed stocks

Report on the source of all seed stocks or other plant material used. If applicable, state the seed stock centre and catalogue number. If plant specimens were collected from the field, describe the collection location, date and sampling procedures.

Novel plant genotypes

Describe the methods by which all novel plant genotypes were produced. This includes those generated by transgenic approaches, gene editing, chemical/radiation-based mutagenesis and hybridization. For transgenic lines, describe the transformation method, the number of independent lines analyzed and the generation upon which experiments were performed. For gene-edited lines, describe the editor used, the endogenous sequence targeted for editing, the targeting guide RNA sequence (if applicable) and how the editor was applied.

Authentication

Describe any authentication procedures for each seed stock used or novel genotype generated. Describe any experiments used to assess the effect of a mutation and, where applicable, how potential secondary effects (e.g. second site T-DNA insertions, mosiacism, off-target gene editing) were examined.

Magnetic resonance imaging

Experimental design

Design type

Resting-state

Design specifications

Subjects were instructed to keep their eyes open and to fixate on a crosshair during the resting-state scans. Each resting-state scan had a duration of 15 min 30 s.

Behavioral performance measures

Following the completion of each resting-state scans subjects had to complete the short New York Cognitive Questionnaire, which includes 12 statemetns about the form and content of the thoughts occurring during the preceding scan. The statemetns are listed in Table 1. For each statement, participants responded with a number between 0 (“Describes my thoughts not at all”) and 100 (“Describes my thoughts completely”).

The Mind-Brain-Body dataset also includes 31 surveys measuring trait-like behaviors from five different categories: personality/habituation behaviors (21 surveys), mind wandering/mindfulness (4 surveys), synesthesia (1 survey), cognitive control/sustain attention (1 survey) and creativity (4 surveys). These surveys were administered only once per subject, as opposed to the experiential surveys that were administered following each resting-state scan (as detailed above). Here, we use these behavioral surveys to evaluate if any of the multiple traits assessed by the available outside-the-scanner behavioral surveys can explain any of the observations we make regarding population differences and prediction accuracy based on FC data. As such, we constrain our investigation to summary survey metrics available for at least 70% of subjects entering these population and predictability FC-based analyses.

Acquisition

Imaging type(s)

Structural and functional

Field strength

3T

Sequence & imaging parameters

Structural: 3D MP2RAGE sequence, voxel size=1.0 mm isotropic, FOV=256×240×176 mm, TR=5000 ms, TE=2.92 ms, TI1=700 ms, TI2=2500 ms, flip angle 1=4°, flip angle 2=5°, bandwidth=240 Hz/Px, GRAPPA acceleration with iPAT factor 3.

Functional Scans: T2*-weighted gradient-echo echo planar imaging (GE-EPI) with multiband acceleration.voxel size=2.3 mm isotropic, FOV=202×202 mm2, imaging matrix=88×88, 64 slices with 2.3 mm thickness, TR=1400 ms, TE=39.4 ms, flip angle=69°, echo spacing=0.67 ms, bandwidth=1776 Hz/Px, partial fourier 7/8, no pre-scan normalization, multiband acceleration factor=4, 657 volumes, duration=15 min 30 s.

Area of acquisition

Whole-brain

Diffusion MRI ☐ Used ☒ Not used

Preprocessing

Preprocessing software

nipype (v. 1.3.2), fsl (v. 5.0.10), freesurfer (v. 5.3.0), ANTs (v. 2.2.0), AFNI, NBS (v1.2), CONN(v22)

Normalization

Spatial normalization was performed using the MPI pipeline publicly available at https://github.com/NeuroanatomyAndConnectivity/pipelines/tree/master/src/lsd_lemon

Normalization template

Data was brought to MNI space using the FSL template MNI152_T1_2mm_brain.nii.gz

Noise and artifact removal

Nuisance regression included the following regressors: motion estimates, first derivative of motion estimates, Legendre polynomials up to 3rd degree, and aCompCorr physiological regressors. Additionally data was bandpass filtered (0.01 - 0.15Hz)

Volume censoring

We used traces of relative displacement to mark as invalid volumes those with a relative displacement above 0.3mm. For any such volume, we also marked as invalid the preceding volume and the following two. Any scan with 30% or more of volumes marked as invalid was discarded from any additional analyses.

Statistical modeling & inference

Model type and settings	Network-based Statistics: Statistical Test=T-test, Threshold=3.1, Component Size=Extent, Permutations=5000, Component Size=Extent, Method=Network Based Statistics.<br><br>Connectome Predictive Modeling: 10-fold cross validation, edge-level threshold p<0.01, run 100 times, prediction performance reported in terms of the Pearson's correlation between observed and predicted values.
Effect(s) tested	We tested for significant differences in functional connectivity between two sample subsets created only on the basis of the experiential reports using network based statistics. To ensure results were not driven by differences in head motion or wakefulness level, we used Mann Whitney U test to confirm there were no significant differences in these two potential confounds across the two subsets. We also used a Wilcoxon Test to make sure there were no differences in terms of age group size across sample sets being compared via Network Based Stastistics.<br><br>Significance of Connectome Predicted Modeling accuracy was tested using 10,000 null permutations in which predictive targets were randomized.

Specify type of analysis: ☐ Whole brain ☒ ROI-based ☐ Both

Anatomical location(s)	We computed representative time-series (spatial mean across voxels) and FC matrices separately for each scan using a modified version the 400ROI / 7 Networks Schaefer atlas (Schaefer, A. et al. Local-Global Parcellation of the Human Cerebral Cortex from Intrinsic Functional Connectivity MRI. Cereb Cortex 28, 3095–3114 (2017)) using the AFNI program 3dNetCorr. Thirteen ROIs were removed from the atlas prior to generating the FC matrices because they include regions suffering from excessive signal dropout. Those correspond to the 12 regions forming the Limbic network and one region part of the default mode network labeled as “LH_Default_Temp_2”. Additionally, 8 subcortical regions for the AAL (Tzourio-Mazoyer, N. et al. Automated Anatomical Labeling of Activations in SPM Using a Macroscopic Anatomical Parcellation of the MNI MRI Single-Subject Brain. Neuroimage 15, 273–289 (2002)) atlas (bilateral thalami, putamen, striatum, and pallidum) were added. Suppl. Fig. 3 shows the final version of the atlas used in the analyses.

Statistic type for inference<br>(See Eklund et al. 2016)	Network size defined in terms of its extent (i.e., number of edges) using Network-based Statistics (Zalesky, A., Fornito, A. & Bullmore, E. T. Network-based statistic: Identifying differences in brain networks. NeuroImage 53, 1197–1207 (2010))
Correction	Functional Connectivity Differences: p<0.05 using 5000 permutations as implemented in version 1.2 of the Network-Based Statistics Toolbox. Correction based on Network size quantified in terms of extent (number of edges). Primary results use an edge-level threshold of p<0.001. Supplementary results use an edge-level threshold of p < 0.005.

Models & analysis

n/a	Involved in the study
☐	☒ Functional and/or effective connectivity
☐	☒ Graph analysis
☐	☒ Multivariate modeling or predictive analysis

Functional and/or effective connectivity	Pearson's Correlation across ROI representative timeseries
Graph analysis	Node degree: number of connections involving a given node/ROI
Multivariate modeling and predictive analysis	Connectome Predictive Modeling (Finn, E., Shen, X., Scheinost, D. & Rosenberg, M. Functional connectome fingerprinting: identifying individuals using patterns of brain connectivity. Nature Neuroscience 18, (2014)). Input features: functional connectivity matrices associated with each individual resting-state scan. Prediction targets: individual answers to each of the 12 short New York Cognition Questionnaire items, and the two low dimensional latent variables found to relate these 12 items. Prediction targets were orthogonalized against head motion estimates prior to training the models.
