# Supplementary material for: In-scanner thoughts contribute to resting-state functional connectivity
Source: Nat Commun. 2026 Jun 27;17:8058. doi: 10.1038/s41467-026-74953-6 (PMC13454257; doi:10.1038/s41467-026-74953-6)
Supplement: Supplementary file 3 [file 41467_2026_74953_MOESM3_ESM.pdf]

nature portfolio

Peer Review File

In-Scanner Thoughts contribute to Resting-state Functional Connectivity

Corresponding Author: Dr Javier Gonzalez-Castillo

This manuscript has been previously reviewed at another journal. This document only contains information relating to versions considered at Nature Communications.

This file contains all reviewer reports in order by version, followed by all author rebuttals in order by version.

Version 0:

Reviewer comments:

Reviewer #3

(Remarks to the Author)
I thank the authors for their attentive and responsive revisions.

These revisions have largely addressed my concerns.

I still believe (like Reviewer 1) that these findings are not especially surprising in light of prior literature. That said, I understand the authors' point that it is necessary and important to directly establish this specific thought-feature-to-resting-functional-connectivity relationship because of its relevance to a broad range of functional connectivity studies, especially in clinical settings.

As a final note: a recent paper in Nature Communications has reported on changes in MTL-DMN functional connectivity in relation to properties of spontaneous thought (Su et al, 2025, Nature Comms; https://www.nature.com/articles/s41467-025-61807-w). I share this only in case the authors believe it is a relevant citation.

Thank you for pushing forward the science of the human mind and brain.

(Remarks on code availability)

Reviewer #4

(Remarks to the Author)
This manuscript investigates whether self-reported experiential content during resting-state fMRI (rsfMRI) accounts for systematic variance in functional connectivity (rsFC). Using 469 scans from the Mind-Brain-Body dataset and retrospective experience reports, the authors identify two dominant thought patterns (TP axes and corresponding scan sets), demonstrate trait-like stability, report widespread rsFC differences between experiential sets using Network-Based Statistics (NBS), and show that rsFC predicts aspects of experience using Connectome-Based Predictive Modeling (CPM). The study addresses an important and timely issue: whether uncontrolled internal mentation during "rest" meaningfully contributes to rsFC variability and reproducibility challenges. However, the current evidence primarily supports an association between trait-like experiential tendencies and rsFC. Stronger within-subject and hierarchical validation analyses, along with threshold robustness checks and clearer methodological reporting, are necessary before firm conclusions about experiential contributions to resting-state connectivity can be drawn. I encourage the authors to further clarify these issues and refine their interpretation in the Results and Discussion sections.

1. While the abstract effectively conveys the importance and potential impact of the findings, its wording appears to slightly overstate the level of inference supported by the analyses presented in the Results and Discussion. Specifically, phrases such as "scans that systematically differ in terms of reported in-scanner experience" and statements emphasizing a "key role" of in-scanner experience in interpreting FC may be read as implying a state-level, potentially causal modulation of FC by

momentary experience. However, much of the evidence presented, particularly the clustering-based comparisons and predictive modeling, could also reflect stable, trait-like experiential tendencies or subject-level differences rather than within-subject state-dependent variation. In contrast, the Discussion adopts a more nuanced and appropriately cautious tone, acknowledging the trait-like stability of experiential reports and the limitations of retrospective measures. To ensure conceptual consistency and avoid overinterpretation, I recommend aligning the abstract more closely with the interpretive scope articulated in the Discussion. Clarifying that the findings demonstrate robust associations between reported experiential patterns and FC, without implying state-level causality, would strengthen the manuscript's internal coherence and interpretational precision.
2. The NBS results report 458 significant edges across 210 nodes (one direction only), suggesting extremely widespread effects. NBS is highly sensitive to edge-level thresholds. Large components may reflect thresholding artifacts or global signal structure.
3. The dataset includes repeated scans per subject. It is essential to confirm that cross-validation folds were partitioned at the subject level. If scans from the same subject appear in both training and test sets, predictive performance may reflect subject fingerprinting rather than experiential prediction.
4. The sNYCQ is retrospective and summarizes experience over the scan. It does not capture temporal fluctuations. Clarify whether sNYCQ reflects overall summary, peak experience, or end-of-scan recall. Explicitly state limits of inference: the study links rsFC to summary-level experiential reports, not time-resolved dynamics.
5. The discussion suggests incorporating experiential measures as covariates to improve reproducibility. Experiential content may constitute part of the clinical phenotype in many populations (e.g., rumination in depression). Automatically regressing it out could remove meaningful variance.

(Remarks on code availability)

Open Access This Peer Review File is licensed under a Creative Commons Attribution 4.0 International License, which permits use, sharing, adaptation, distribution and reproduction in any medium or format, as long as you give appropriate credit to the original author(s) and the source, provide a link to the Creative Commons license, and indicate if changes were made.
In cases where reviewers are anonymous, credit should be given to 'Anonymous Referee' and the source.
The images or other third party material in this Peer Review File are included in the article's Creative Commons license, unless indicated otherwise in a credit line to the material. If material is not included in the article's Creative Commons license and your intended use is not permitted by statutory regulation or exceeds the permitted use, you will need to obtain permission directly from the copyright holder.
To view a copy of this license, visit https://creativecommons.org/licenses/by/4.0/

Reviewer #3 (Remarks to the Author):

I thank the authors for their attentive and responsive revisions.

These revisions have largely addressed my concerns.

I still believe (like Reviewer 1) that these findings are not especially surprising in light of prior literature. That said, I understand the authors' point that it is necessary and important to directly establish this specific thought-feature-to-resting-functional-connectivity relationship because of its relevance to a broad range of functional connectivity studies, especially in clinical settings.

We thank the reviewer for taking the time to go over this revised version of the manuscript and for acknowledging the importance of our work for clinical settings.

As a final note: a recent paper in Nature Communications has reported on changes in MTL-DMN functional connectivity in relation to properties of spontaneous thought (Su et al, 2025, Nature Comms; https://www.nature.com/articles/s41467-025-61807-w). I share this only in case the authors believe it is a relevant citation.

We thank the reviewer for bringing this relevant work to our attention. We now reference it in the introduction (line 58) as one more example of prior work looking at aspects of spontaneous thoughts during rest.

"A few instances where such data is available suggest that some aspects of in-scanner experience can be related to resting-state signals. For example, … stronger connectivity between the default mode network and the control networks has been linked to reduced variability in the semantic content of thoughts[10]; and the qualia of inner voices (i.e., inner hearing vs. inner speaking) was associated…".

We also mention it later as an example of an experimental paradigm that permits exploring state-level relationships between momentary aspects of experience and concurrent brain activity/connectivity:

"Such state-level questions would require temporally resolved experiential measurements, such as intermittent in-scanner probes[36] or continuous thought reporting[10], which are not available in this dataset."

Thank you for pushing forward the science of the human mind and brain.

Thank you for the very kind words!

<u>Reviewer #4 (Remarks to the Author):</u>

This manuscript investigates whether self-reported experiential content during resting-state fMRI (rsfMRI) accounts for systematic variance in functional connectivity (rsFC). Using 469 scans from the Mind-Brain-Body dataset and retrospective experience reports, the authors identify two dominant thought patterns (TP axes and corresponding scan sets), demonstrate trait-like stability, report widespread rsFC differences between experiential sets using Network-Based Statistics (NBS), and show that rsFC predicts aspects of experience using Connectome-Based Predictive Modeling (CPM). The study addresses an important and timely issue: whether uncontrolled internal mentation during "rest" meaningfully contributes to rsFC variability and reproducibility challenges. However, the current evidence primarily supports an association between trait-like experiential tendencies and rsFC. Stronger within-subject and hierarchical validation analyses, along with threshold robustness checks and clearer methodological reporting, are necessary before firm conclusions about experiential contributions to resting-state connectivity can be drawn. I encourage the authors to further clarify these issues and refine their interpretation in the Results and Discussion sections.

We thank the reviewer for their input. We agree that this work only demonstrates "*an association between trait-like experiential tendencies and rsFC*", which reflects the limitations of the data itself. Since this dataset does not include measures of within-subject changes in cognitive states over time, it cannot be used to explore one-to-one links between momentary experience and concurrent functional connectivity. In the revised manuscript, we further emphasize this limitation through added text in the abstract, introduction and discussion to more explicitly state this.

New abstract: please see response to item 1 in the next page.

New text added to the introduction:

"Here, we answer these three questions affirmatively, using a subset of 469 resting-state scans from the publicly available Max Planck Institute Mind-Brain-Body dataset[35]. These scans are annotated with retrospective debriefing self-reports about in-scanner thoughts and wakefulness levels (Table 1). Although limited in scope, these annotations provide rare and valuable information about participants' subjective experience during rsfMRI acquisition. Given the retrospective nature of these experiential measures, however, the present analyses focus exclusively on summary dimensions of in-scanner experience, such as participants' general tendency to think in images or to engage in inner speech. They do not test whether momentary experiential events, such as imagining a walk through the woods during light rain, are associated with concurrent fluctuations in brain

activity. Such state-level questions would require temporally resolved experiential measurements, such as intermittent in-scanner probes[36] or continuous thought reporting[10], which are not available in this dataset."

We have also added a modifier to the opening sentence of the discussion, as follows:

"*This investigation provides strong evidence for the key—yet often overlooked—role that trait-like aspects of the subjective in-scanner experience plays in resting-state FC.*"

And the following text has been added to the Online Methods:

"*Upon completion of each resting-state scan, subjects completed the sNYCQ, a 12 questions survey that gathers information about different aspects of the form and content of thoughts incurred during the immediately preceding resting-state scan. Because the sNYCQ is collected immediately after each run and retrospectively queries the preceding measurement period, it provides a summary-level characterization of in-scanner experience rather than a temporally resolved account of within-run experiential fluctuations.*"

1. While the abstract effectively conveys the importance and potential impact of the findings, its wording appears to slightly overstate the level of inference supported by the analyses presented in the Results and Discussion. Specifically, phrases such as "scans that systematically differ in terms of reported in-scanner experience" and statements emphasizing a "key role" of in-scanner experience in interpreting FC may be read as implying a state-level, potentially causal modulation of FC by momentary experience. However, much of the evidence presented, particularly the clustering-based comparisons and predictive modeling, could also reflect stable, trait-like experiential tendencies or subject-level differences rather than within-subject state-dependent variation. In contrast, the Discussion adopts a more nuanced and appropriately cautious tone, acknowledging the trait-like stability of experiential reports and the limitations of retrospective measures. To ensure conceptual consistency and avoid overinterpretation, I recommend aligning the abstract more closely with the interpretive scope articulated in the Discussion. Clarifying that the findings demonstrate robust associations between reported experiential patterns and FC, without implying state-level causality, would strengthen the manuscript's internal coherence and interpretational precision.

We thank the reviewer for this important comment. We have now revised the abstract to clearly convey that the focus of this work is on trait-level aspects of experience. We also explicitly state that we do not investigate relationships to momentary experience. Here is the updated abstract (changes highlighted in red):

“Resting-state fMRI (rsfMRI) scans—acquired in the absence of experimentally controlled stimuli or task demands—are widely used to identify aberrant patterns of functional connectivity (FC) in clinical populations. To minimize interpretational uncertainty, researchers routinely control for across-cohort disparities in age, gender, co-morbidities, and head motion. Yet, studies rarely consider the possibility that systematic differences in inner experience (i.e., how subjects think and feel during the scan) directly affect FC measures. Here, using a rsfMRI dataset comprising 469 scans with retrospective experiential annotations, we show that summary descriptors of in-scanner experience are reproducible across visits and subject-specific, consistent with trait-like characteristics. We further show that wide-spread significant differences in FC are observed between scans that are associated with different reported experiential profiles, and that FC can predict specific experiential dimensions with performance comparable to that reported for demographic, cognitive, and clinical variables. Together, these findings highlight the key role that in-scanner experience should play when interpreting FC in the context of rsfMRI. Given the available experiential measures are retrospective summaries, these results speak to stable experiential tendencies rather than potential moment-to-moment, state-dependent relationships between ongoing experience and concurrent brain activity.”

2. The NBS results report 458 significant edges across 210 nodes (one direction only), suggesting extremely widespread effects. NBS is highly sensitive to edge-level thresholds. Large components may reflect thresholding artifacts or global signal structure.

The reviewer is correct that NBS results can be sensitive to the choice of edge-level threshold. To evaluate the robustness of our findings, we have now repeated the analysis using a second, less stringent edge-level threshold ($p < 0.005$), while keeping the network-level threshold unchanged. These additional results are reported in the revised manuscript and shown in Supplementary Figure 7.

Edge-Level p<0.005 | Network-Level p_{FWE}<0.05 | Differences in Functional Connectivity across sets [Set A > Set B]

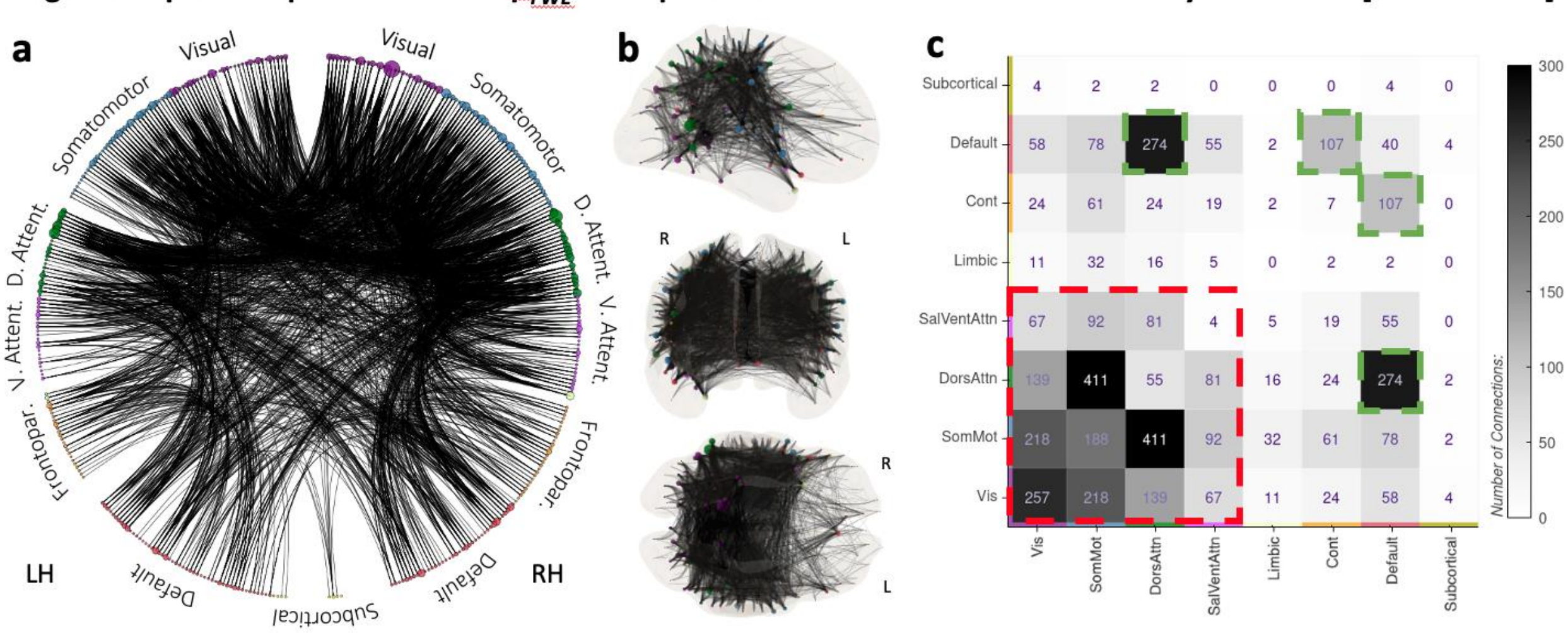

Supplementary Figure 7 | Significant differences in FC across scans with different thought patterns based on a less restrictive edge-level threshold. a, Circos plot with connections rendered as significant (NBS, 5000 Permutations, Edge-Level Threshold $p < 0.005$, Network-Level Threshold $p < 0.05$) for the Set A > *Set B* contrast. In this plot, ROIs are depicted as colored dots (color indicating network membership) on a circumference, and significant connections are depicted as black lines. ROIs are grouped by network and hemisphere, with left hemisphere ROIs on the left, and right hemisphere ROIs on the right of the plot. The size of the dot is proportional to the degree of the ROI (i.e., number of significant connections emerging from the ROI). **b,** Same information as in a, but this time on a glass brain. ROI size denotes degree and ROI color denotes network membership. **c,** Counts of between- and within-network connections significant for the contrast *Set A > Set B*.

The following text has been added to the results section:

"FC matrices for all scans in the A and B sets were created using the 400 ROI/7 network Schaefer Atlas[46], modified to also include 8 subcortical ROIs from the AAL Atlas[47] (Fig. 3a, Suppl. Fig. 3). Significant differences in rsFC across these two sets were evaluated using Network-Based Statistics[48] (NBS; $p_{FWE}<0.05$)… Because NBS results can be sensitive to the choice of edge-level threshold, we repeated the analyses using a less restrictive edge-level threshold of $p < 0.005$, while keeping the network-level threshold unchanged ($p_{FWE} < 0.05$). This threshold yielded a larger significant component, comprising 2,339 connections across 342 nodes. Despite this increase, the distribution of significant connections across the eight networks was qualitatively similar to that observed in the primary analysis (Suppl. Fig. 7). The laterality index also remained negative, consistent with right-lateralized effects under this less restrictive edge-level threshold ($FC_{laterality\ index} = -0.27$)."

3. The dataset includes repeated scans per subject. It is essential to confirm that cross-validation folds were partitioned at the subject level. If scans from the same subject appear in both training and test sets, predictive performance may reflect subject fingerprinting rather than experiential prediction.

We agree with the reviewer that this is a particularly important methodological consideration, specifically given the trait-like nature of the studied experiential aspects. Cross-validation was indeed performed considering subject identity. We already stated this in the methods as follows:

"Given that our dataset includes multiple scans per subject, it was important to consider subject identity during the split of the data into training and testing sets. When building folds, we ensured that all scans from the same subject were always included in the testing or training sets, but not across both. In other words, we did not use information from scans of one subject to predict a behavioral score for a different scan from the same subject."

That said, and because we agree with the reviewer that this needs to be clearly stated (and exemplified), we have now also conducted CPM analyses in which subject identity is not considered when assigning scans to the training and test sets. As predicted by the reviewer, this leads to a substantial inflation of prediction accuracy, likely because of the

demonstrated trait-like, fingerprinting-like nature of retrospectively measured in-scanner experience. For that reason, we have decided to include these results as an additional supplementary figure and table:

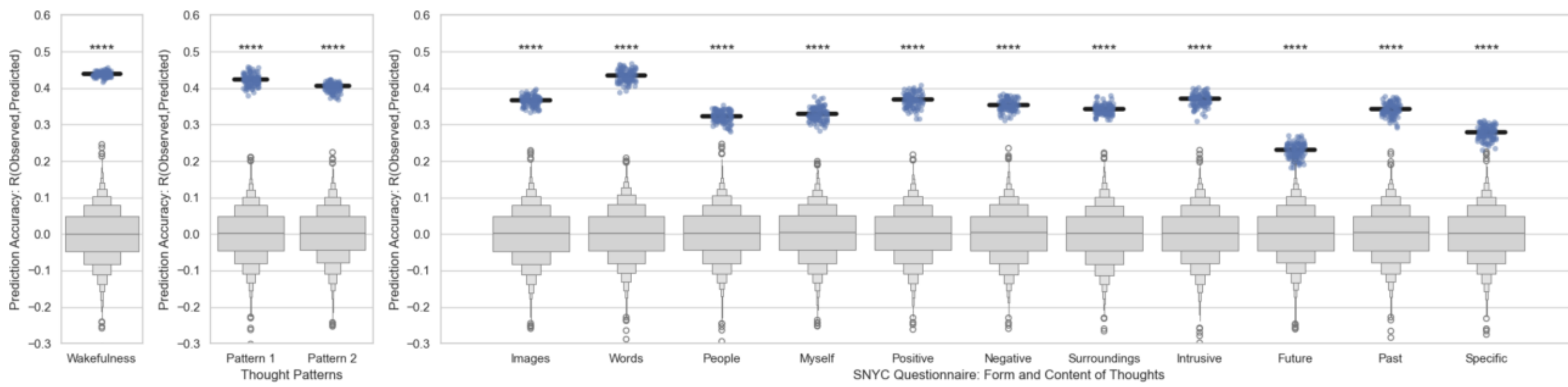

Supplementary Figure 4 | Prediction Accuracy for scan-level cross-validation CPM analyses. **a,** Answers to item *"I was completely awake"*. **b,** *Thought Pattern 1* and *Thought Pattern 2* as defined using the ICQF algorithm. **c,** Answers to the 11 items about the form and content of in-scanner thoughts. In all plots, the gray boxenplot depicts the null distribution of accuracy values across 10,000 randomized permutations. Blue dots correspond to the Pearson's correlation between observed and predicted values (i.e., prediction accuracy) in each of the 100 iterations used to predict each target. Bold black lines represent the median prediction accuracy across the 100 iterations. Significance annotations correspond to the non-parametric, uncorrected p-value of the median prediction accuracy according to each null distribution (**** *p<0.0001,* *** *p<0.001,* ** *p<0.01,* * *p< 0.05*).

This is the new text in the results section introducing the new supplementary figure and table:

"As the dataset includes repeated scans from the same participants, our primary analyses used subject-level cross-validation folds. Specifically, all scans from a given participant were assigned to the same fold within each cross-validation iteration, ensuring that no participant contributed scans to both the training and test sets. Under this more conservative procedure, CPM significantly predicted reported wakefulness levels, TP1 and TP2 factor scores, and responses to the Images, Surroundings, and Past questionnaire items ($p_{non-parametric, uncorrected} < 0.05$; Fig. 5a–c; Table 4). Of these, "Past" did not survive multiple comparison correction based on the False Discovery Rate (FDR[52]) method ($p_{non-parametric,FDR}<0.05$).

For comparison, we also repeated the analyses using scan-level cross-validation folds, in which subject identity was not constrained and scans from the same participant could appear in both the training and test sets. Under this less conservative procedure, all 14 experiential indices were significantly predicted from rsFC (Suppl. Fig. 4). Because this approach allows subject-specific information to contribute to prediction accuracy, we do not interpret these secondary results as additional evidence that rsFC contains information about in-scanner experience. Rather, they illustrate how dependencies among repeated observations from the same individuals can inadvertently inflate predictive performance of traits. Accordingly, all subsequent results regarding successful CPM models correspond to the subject-level cross-validation analyses."

This is the new text in the online methods section describing this alternative cross-validation scheme:

"*We repeated this whole process 100 times to ensure our results were robust to variability in how the data was split into training and testing folds during the cross-validation step. Given that our dataset includes multiple scans per subject, it was important to consider subject identity during the split of the data into training and testing sets. For the primary analyses, when building folds, we ensured that all scans from the same subject were always included in the testing or training sets, but not across both. In other words, we did not use information from scans of one subject to predict a behavioral score for a different scan from the same subject. We refer to these results as subject-level cross-validation CPMs throughout the manuscript.*

Additionally, and as secondary analyses, we also report results using a scan-level cross-validation scheme in which subject identity is not considered when building cross-validation sets. In this case, scans from the same subject could appear in both sets. Results from these secondary CPM analyses are only reported as supplementary materials to emphasize the importance of considering repeated measures when prediction targets behave primarily as traits."

4. The sNYCQ is retrospective and summarizes experience over the scan. It does not capture temporal fluctuations. Clarify whether sNYCQ reflects overall summary, peak experience, or end-of-scan recall. Explicitly state limits of inference: the study links rsFC to summary-level experiential reports, not time-resolved dynamics.

This work relies on a publicly available dataset (the Brain-Mind-Body dataset) and on the information provided in the accompanying *Scientific Data* publication by Mendes et al. That publication states that the sNYCQ was administered at the end of each resting-state scan and that subjects were instructed to answer each item on a scale from 0 ("describes my thoughts not at all") to 100 ("describes my thoughts completely"). No additional explicit information directly addressing the reviewer's concern is provided in that source.

That said, related papers by some of the co-authors of Mendes et al. that use questionnaires such as the sNYCQ to capture in-scanner experience provide additional relevant context. In particular, Gorgolewski et al. (2014), on which Mendes is also a co-author, provides a link to a GitHub repository containing the code used to administer the questionnaire. The instructions presented to participants there read as follows: "We are interested in the thoughts and feelings that you experienced during the MRI. Please indicate which statement describes the thoughts that you experienced while completing the MRI. Indicate the extent to which your thinking corresponded to each item by selecting the relevant option associated with each sentence." Based on this information, we interpret the sNYCQ as reflecting a retrospective summary of experience across the entire, immediately preceding resting-state scan.

To make this point explicit, we have added the following text on page 24 of the Online Methods to clarify that the sNYCQ captures summary-level reports rather than time-resolved dynamics:

"Upon completion of each resting-state scan, subjects completed the sNYCQ, a 12-question survey that gathers information about different aspects of the form and content of thoughts experienced during the immediately preceding resting-state scan. Because the sNYCQ is collected immediately after each run and retrospectively queries the preceding fMRI measurement period, it provides a summary-level characterization of in-scanner experience rather than a temporally resolved account of within-run experiential fluctuations"

5. The discussion suggests incorporating experiential measures as covariates to improve reproducibility. Experiential content may constitute part of the clinical phenotype in many populations (e.g., rumination in depression). Automatically regressing it out could remove meaningful variance.

During the previous round of review, a similar concern was raised by one of the reviewers. In response, we added clarifying text to the final paragraph of the Introduction. However, we agree that this point would benefit from further refinement, and we have revised the text accordingly.

To address this unresolved concern, we have now modified the text in the intro as follows:

"This is a relatively simple addition, compared to the cost and complexity of executing a single resting-state scan, and would enrich resting-state datasets with new opportunities for analysis and discovery. For example, experiential measures could be incorporated into statistical models in a manner analogous to other individual-difference variables, depending on the scientific question. Doing so may improve the accuracy and interpretability of rsfMRI-based predictive models and help uncover new brain–behavior relationships. However, as with any covariate, these measures should not be treated as nuisance variables by default. Care is needed to avoid inadvertently removing variance of substantive interest, particularly when specific in-scanner experiential profiles or patterns of thought may be closely related to the clinical phenotype under study (e.g., rumination in depression). Similarly, such richer…"

We have also modified the discussion as follows:

"An alternative approach would be to collect experiential information using instruments such as the sNYCQ or the ARSQ[54,56], so that these measures can be incorporated into analytical models ~~(similar to how motion and other physiological measures are often incorporated in rsfMRI studies)~~. However, their inclusion requires careful consideration. Treating experiential measures as nuisance covariates by default could remove meaningful variance when in-scanner experience is related to the phenotype or process

under investigation, as may be the case for rumination in depression. Conversely, when experiential variability is not itself the target of interest, modeling these measures may help reduce inter-individual variability that could otherwise obscure other sources of rsFC variance, including variance associated with other experience-independent neural processes occurring during rest. Accordingly, experiential measures should be incorporated in a hypothesis-driven manner, rather than automatically regressed out."
